# Colloid Transport in Bicontinuous Nanoporous Media

**DOI:** 10.1021/acs.langmuir.4c00037

**Published:** 2024-05-17

**Authors:** Aoyan Liang, Chang Liu, Paulo S. Branicio

**Affiliations:** Mork Family Department of Chemical Engineering and Materials Science, University of Southern California, Los Angeles, California 90089-0242, United States

## Abstract

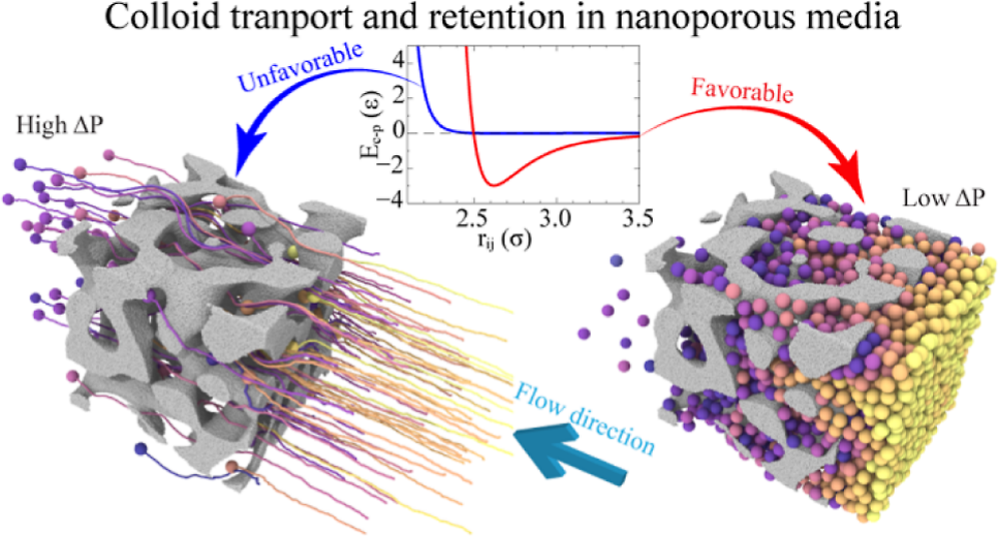

Colloid transport and retention in porous media are critical processes influencing various Earth science applications, from groundwater remediation to enhanced oil recovery. These phenomena become particularly complex in the confined spaces of nanoporous media, where strong boundary layer effects and nanoconfinement significantly alter colloid behavior. In this work, we use particle dynamics models to simulate colloid transport and retention processes in bicontinuous nanoporous (BNP) media under pressure gradients. By utilizing particle-based models, we track the movement of each colloid and elucidate the underlying colloid retention mechanisms. Under unfavorable attachment conditions, the results reveal two colloid retention mechanisms: physical straining and trapping in low-flow zone. Furthermore, we investigate the effects of critical factors including colloid volume fraction, *d*, pressure difference, Δ*P*, interaction between colloids and BNP media, *E*_c–p_, and among colloids, *E*_c–c_, on colloid transport. Analysis of breakthrough curves and colloid displacements demonstrates that higher values of *d*, lower values of Δ*P*, and strong *E*_c–p_ attractions significantly increase colloid retention, which further lead to colloid clogging and jamming. In contrast, *E*_c–c_ has minimal impact on colloid transport due to the limited colloid–colloid interaction in nanoporous channels. This work provides critical insights into the fundamental factors governing colloid transport and retention within stochastic nanoporous materials.

## Introduction

Colloids are particles typically sized between 1 and 1000 nm, including engineered nanoparticles, microbes, fine powders, and clay.^1^ Their transport and retention processes within porous media are involved in a wide range of natural and engineering processes, such as water and wastewater filtration, groundwater remediation, hydrocarbon recovery, and drug delivery.^2−5^ Therefore, modeling and predicting the fate and transport of colloids in porous media are of fundamental interest in various fields, spanning industrial manufacturing, environmental engineering, medical, and Earth sciences.^6−8^

A widely used mechanistic modeling approach for predicting colloid transport in saturated porous media is colloid filtration theory (CFT).^5,9^ CFT provides reasonably accurate predictions for colloid transport in porous media under favorable attachment conditions.^6,10^ Such conditions occur when colloids and collectors possess opposite charges, leading to electrostatic attractive forces that significantly influence transport dynamics. In this context, a collector refers to the solid phase or surface within a porous medium that captures or interacts with colloidal particles as they pass through the medium. However, substantial disparities arise between CFT predictions and empirical observations under unfavorable attachment conditions when colloid-collector repulsion is large.^11,12^ These discrepancies can be attributed to the neglect of surface roughness, charge heterogeneity, and intricate porous geometries in CFT.^13−17^ Although the incorporation of the attachment efficiency, α, improves CFT predictability under unfavorable attachment conditions, the unpredictable and ungeneralizable nature of α hinders broader CFT applications.^18,19^ Moreover, CFT predictions are challenging when considering the concurrent influences of multiple transport mechanisms.^8^

Continuum modeling is another commonly used prediction method, which have proven to be suitable for the description of colloid transport in porous media in large spatial scales ranging from laboratory column experiments to aquifer.^20−25^ Nonetheless, the assortment of model equations proposed by various researchers and the lack of efficient validation methods limit a reliable and comprehensive application in the prediction of colloid transport and retention.^8^ Furthermore, the lack of pore-scale descriptions in continuum models makes them inadequate for simulating colloid transport through nanoporous media and introduces uncertainty in linking the observed colloid breakthrough curves (BTCs) to fundamental underlying processes.^6,26,27^

Recently, reports on the abnormal fluid behavior under nanoconfinement and the boost of hydrocarbon production from unconventional tight reservoirs have garnered significant interest to nanoconfined fluid and colloid flow.^28−31^ In contrast to coarse-grained porous media, unconventional reservoirs are characterized by nanoscale pores (e.g., pore sizes < 50 nm) and heterogeneous porous formations.^32,33^ Such a nanoporous structure leads to a strong nanoconfinement effect and low permeability that markedly heightens the challenge of accessing untapped hydrocarbon resources and diminishes its production rate.^34,35^ In addition, the properties and behavior of nanoconfined fluids and colloids exhibit deviations from the predictions from traditional mechanistic and continuum modeling approaches due to significant boundary layer effects.^36^ Therefore, novel theoretical modeling tools are needed to gain a comprehensive understanding of fluid and colloid flow within nanoporous media, particularly considering the influence of nanoconfinement and boundary layer effects.

Many studies attempted to address this problem by using molecular dynamics (MD) simulations to investigate the behavior of nanoconfined water and hydrocarbons.^37−44^ While MD simulations provide remarkable accuracy at the nanoscale, their computational demands are often excessive.^45,46^ On the other hand, the dissipative particle dynamics (DPD) method, originally proposed by Hoogerbrugge and Koelman,^47^ offers an alternative approach to address these challenges. Comparing with MD simulations and the Lattice Boltzmann method, the DPD method is able to bridge the gap between capturing nanoconfinement effects and maintaining computational feasibility for the larger systems and extended time scales.^33,48^ In particular, DPD’s soft interaction potentials and coarse-grained nature facilitate simulations over larger spatiotemporal scales compared to MD, thus providing a balance between efficiency and detail that is particularly advantageous for exploring the interaction dynamics.^49,50^ Moreover, DPD accurately describes colloid transport and retention processes across a wide range of conditions since fluid flow and colloid transport are fully coupled in the DPD method.^51^ Numerous studies have applied and validated DPD and its variant model to investigate the fluid behavior under nanoconfinement,^49,52−57^ as well as colloid suspension flow.^51,58^ For example, Dzwinel et al.^59^ compared several modeling approaches for simulating mesoscopic dynamics of colloid suspensions and indicated that the DPD framework offers a realistic representation of molecular physics. Chatterjee et al.^60^ utilized a similar method to model colloid suspensions with high-viscosity solvents, aligning well with experimental results. Pan and Tartakovsky^51^ showcased the application of the DPD method in modeling colloid transport through the Happel sphere-in-cell porous media, evidencing its effectiveness in mirroring the interplay between fluid dynamics and colloid behavior. Rao et al.^61^ demonstrated the adaptability and accuracy of an enhanced DPD model in capturing both the static and dynamic properties of complex fluids such as hydrocarbons and water. Nevertheless, research on colloid transport behavior within stochastic nanoporous media remains limited, particularly regarding the intricacies of colloid–colloid and colloid–media interactions, which still require comprehensive understanding.

In this work, we focus on understanding the colloid transport and retention processes within fine-grained stochastic bicontinuous nanoporous (BNP) media, where the influence of boundary layer is significant. Such processes are simulated through particle dynamics models. We investigate the colloid retention mechanisms and the impact of four critical factors that govern the dynamics of colloid transport in nanoporous media, i.e., colloid volume fraction, *d*, pressure difference, Δ*P*, colloid–BNP interaction, *E*_c–p_, and colloid–colloid interaction, *E*_c–c_. This study sheds light on the underlying mechanisms and factors that dictate the interplay of colloid transport within stochastic nanoporous media.

## Methods

DPD is a mesoscale model that characterizes fluid dynamics using coarse-grained atom clusters represented as DPD particles while preserving essential molecular interactions. In the conventional DPD model, DPD particles interact via pairwise forces, ***F***_*ij*_, that comprise three distinct components, named conservative force ***F***_*ij*_^C^, dissipative force ***F***_*ij*_^D^, and random force ***F***_*ij*_^R^.
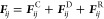
1***F***_*ij*_^C^ is controlled by a conservative force coefficient *A* and a weight function *w*(*r*_*ij*_) as
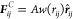
2where *r*_*ij*_ denotes the distance between particle *i* and particle *j*, *r*_*ij*_ = |***r***_*i*_ – ***r***_*j*_|, and ***r*^**_*ij*_ is the unit vector pointing from particle *i* to particle *j*, ***r*^**_*ij*_ = ***r***_*ij*_/*r*_*ij*_. The term *w*(*r*_*ij*_) is a linear function varying from 0 to 1 defined as
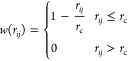
3where *r*_c_ is the cutoff distance. The dissipative force ***F***_*ij*_^D^ and random force ***F***_*ij*_^R^ are defined as
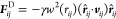
4
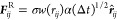
5where ***v***_*ij*_ = ***v***_*i*_ – ***v***_*j*_ is the velocity difference between particle *i* to particle *j*; Δ*t* is the simulation time step; and α is a random value for each *i*, *j* pair that is chosen from a Gaussian distribution with zero mean and unit variance. γ and σ are the dissipative and random coefficients, respectively. They satisfy a fluctuation–dissipation theorem, i.e., , where *k*_B_ is the Boltzmann constant and *T* is the temperature of the system.

However, conventional DPD can only simulate the fluid behavior in confined spaces due to its intrinsic pure repulsive force.^62,63^ To address this weakness, many-body DPD (mDPD), as a modification to the original DPD model, was proposed by introducing an attractive term and a density-dependent repulsive force term into the conservative force.^64^ In the mDPD model, the conservative force ***F***_*ij*_^C^ is composed of a density-independent long-range attractive term *A*_*ij*_*w*(*r*_*ij*_)***r*^**_*ij*_ and a density-dependent short-range repulsive term *B*_*ij*_(ρ̅_*i*_ + ρ̅_*j*_) *w*_d_(*r*_*ij*_)***r*^**_*ij*_
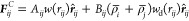
6where *A*_*ij*_ < 0 is the attractive force coefficient and *B*_*ij*_ > 0 is the repulsive force coefficient and ρ̅_*i*_ and ρ̅_*j*_ are the average local density at the positions of particles *i* and *j*, respectively. *A_ij_* and *B*_*ij*_ are dependent on the types of particles *i* and *j*. The repulsive term has a shorter cutoff distance *r*_d_ in its weight function *w*_d_ (*r*_*ij*_) than that in the attractive term, i.e., *r*_d_ < *r*_c_, to accurately describe a stable liquid–vacuum interface.^64^*w*_d_ (*r*_*ij*_) is defined as
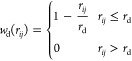
7Following previous work,^54^ we choose *r*_d_ to be 0.75*r*_c_, which provides a reasonable equation of state.^53,65^ ρ̅_*i*_ is defined as
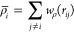
8
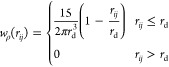
9where the normalized weight function *w*_ρ_(*r*_*ij*_) satisfies ∫_0_^∞^4π*r*^2^*w*_ρ_(*r*) d*r* = 1. This modification makes mDPD well-suited for handling multiphase systems such as systems containing liquid–liquid, liquid–vapor, and liquid–vacuum interfaces.^49^ Also, the flexible parametrization in mDPD models allows the modeling for various fluids.^61^

In this work, we consider both fluid and solid phases as DPD particles. The solid phase consists of the BNP and pistons. The fluid–solid and fluid–fluid interactions are described by the mDPD model. Following previous work on fluid flow in nanoporous media conducted by Xia et al.^52^ and Liu and Branicio,^54^ the mDPD parameters used for both interactions are γ = 4.5 ετ/σ^2^, *B* = 25 ε/σ, *r*_d_ = 0.75 σ, and *r*_c_ = 1.0 σ, where ε, τ, and σ are the reduced energy, time, and length units, respectively. We use *A* = −40 ε/σ for fluid–fluid interactions and −35 ε/σ for fluid–solid interactions. This parameter selection offers reasonable surface tension and wettability. For simplicity, we consider the solid phases as nondeformable rigid bodies and the BNP structure as stationary. Therefore, solid–solid interactions are disregarded.

**Figure 1:**
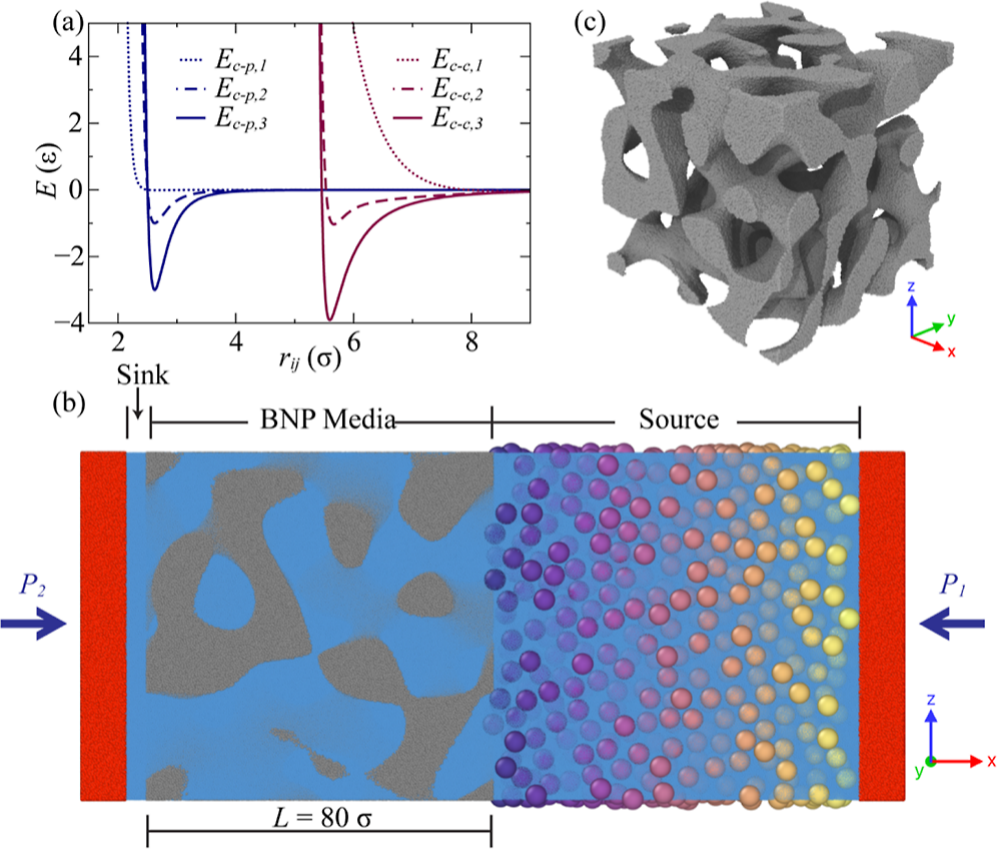
Illustrations of BNP media and particle dynamics simulation models and relevant interactions. (a) Interaction potential energy curves between colloids and BNP media particles, *E*_c–p_, and between colloids, *E*_c–c_, with respect to separation distance. (b) Simulation model with colloid volume fraction, *d* = 0.30, in the initial configuration. Blue-, gray-, and red-colored particles represent particles in the fluid, BNP media, and pistons, respectively. The larger sphere particles with gradient color represent colloids with a diameter of 5 σ. (c) BNP media morphology employed in all models.

There are several methods to incorporate the colloidal particles into the DPD system. Some early studies on colloid suspensions modeled colloidal particles as “packets” of DPD particles moving as rigid bodies.^66−68^ For example, soft particles such as red blood cells have been modeled by clusters of DPD particles to simulate the membrane and internal structure,^69−73^ with cell–cell interactions represented through specific intergranular potentials.^74,75^ Although this method provides versatility in accommodating different colloid shapes, it could potentially result in notable penetration of fluid particles into colloidal particles due to the soft DPD interaction.^50^ Moreover, considering a colloid as a cluster of DPD particles significantly increases the unnecessary computational cost for spherical colloids, especially when the colloid volume fraction is high. On the other hand, colloids can be modeled as individual spherical particles by introducing stiff conservative interactions between colloids and DPD particles such as exponential conservative forces^51,58,76^ and Lennard–Jones (L–J) interactions.^50,59,60,77,78^ This method has demonstrated better capability in capturing accurate hydrodynamic interactions.^50^ In this work, we assume that the colloid rigidity and deformation resistance are significant, which is a modeling assumption particularly relevant to the simulation of nonflexible colloids such as nanoparticles. We expect that this simulation method is appropriate in various length scales as long as the colloids exhibit substantial rigidity and deformation resistance under fluid flow. In this scenario, we employ a shifted L–J potential to model colloids as rigid spherical particles and focus on the effect of colloid–colloid and colloid–BNP interactions. The shifted potential is a modification of the original L–J potential, designed to prevent fluid DPD particles from penetrating into the colloid by ensuring the potential diverges at a nonzero particle separation. This is achieved by adding a *δ*_LJ_ term to the original L–J formula
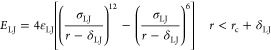
10where *ε*_LJ_ is the energy well depth that measures the strength of the attraction between colloids and DPD particles, *σ*_LJ_ is a parameter that controls the size of the colloid, *r* is the distance separation between colloids and DPD particle centers, *δ*_LJ_ is a parameter that shifts the potential, and *r* + *δ*_LJ_ is the cutoff distance. When *r* = *σ*_LJ_ + *δ*_LJ_, *E*_LJ_ is zero. By fine-tuning the parameters *σ*_LJ_ and *δ*_LJ_, we can simulate rigid colloids of specific sizes, while adjustments to *ε*_LJ_ allow us to control the strength of the interaction between colloids and the nanoporous structure. Here, we choose *σ*_LJ_ = 1 σ and *δ*_LJ_ = 1.5 σ to represent a hard-sphere-like colloid with a radius of 2.5 σ. For the colloid–fluid and colloid–piston interactions, we use a constant *ε*_LJ_ = 1 ε. For the colloid–BNP interaction, namely, *E*_c–p_, we vary the value of *ε*_LJ_ to study the impact of interaction strength between colloid and BNP on colloid transport behavior. The colloid–BNP interactions employed in this work are shown as the blue curves in Figure 1a. Three interactions, *E*_c–p,1_, *E*_c–p,2_, and *E*_c–p,3_, represent the weakest, intermediate, and the strongest attractions between colloids and BNP particles, with *ε*_LJ_ = 0.01, 1, and 3 ε, respectively.

For colloid–colloid interactions, we employ the Derjaguin–Landau–Verwey–Overbeek (DLVO) interaction combining van der Waals interaction and electrostatic repulsion.^79,80^ The van der Waals interaction consists of an attraction term, *E*_A_, and a repulsion term *E*_R_ given by the following equations
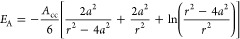
11
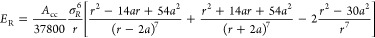
12where *A*_cc_ is the Hamaker constant,^81^*a* is the radius of the colloid, which is set at 2.5 σ in our simulations, *σ*_R_ is a repulsive scaling factor, and *r* is the distance separation between two colloids. This equation is derived from treating each colloidal particle as an integrated collection of L–J particles proposed by Everaers and Ejtehadi.^82^ Following typical values used in previous work,^50,82,83^ we set values for *A*_cc_ and *σ*_R_ as 39.5 ε and 1.0 σ, respectively.

The electrostatic repulsion part, *E*_E_, is derived from the Coulombic interaction between two colloids screened due to the presence of an electrolyte.^83^ The formula is defined as
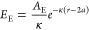
13where *A*_E_ is a measurement of the magnitude of electrostatic repulsion and κ is the inverse Debye screening length. Thus, the final colloid–colloid interaction, *E*_c–c_, is given by
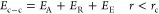
14where *r*_c_ is the cutoff distance.

Due to the inherent generality of the DPD model, the selection of parameters is somewhat arbitrary and primarily aimed at exploring colloid transport under a variety of physicochemical scenarios. Here, we use a constant *κ* = 2 σ^–1^ and adjust *A*_E_ value to achieve different interaction strengths between colloids. The colloid–colloid interactions used in this work are shown as the dark red curves in Figure 1a. Three interactions, *E*_c–c,1_, *E*_c–c,2_, and *E*_c–c,3_, represent the weakest, intermediate, and the strongest attractions between colloids, with *A*_E_ = 0, 20, and 100 ε/σ, respectively. The interaction framework employed in this work is similar to the two-level discrete-particle model proposed by Dzwinel and Yuen,^78^ and its efficacy has been demonstrated through its successful application in many studies on modeling the rheology of colloidal suspensions.^50,59,60,77^ This model captures the essential behavior of colloids, including their navigation around obstacles and interaction dynamics with fluid and collector. Within the scope of our simulations, the fluid DPD particles are configured to be significantly smaller than the colloidal particles, thereby typically facilitating the fluid particles to navigate “around” the colloids. These interactions, along with the hydrodynamic effects, are naturally captured in the pairwise dissipative forces within the DPD framework. This modeling approach ensures that the colloid interactions with both solid and fluid particles are realistically simulated.^51,84^ While DPD particles themselves are not direct analogs of physical entities, the model’s parameters can be carefully calibrated to simulate specific soft matter behaviors like water and heptane.^53,61^

An illustration of the simulation system is shown in Figure 1b. The system consists of four types of particles, i.e., fluid, BNP, piston, and colloid particles. Two fluid reservoirs, sink and source, are placed adjacent to the BNP media, where the fluid is confined by two mobile pistons. The colloid and fluid are made to move between source and sink regions through nanoporous media by the coordinated movement of two pistons. While the detailed process involved in the generation of a system containing fluid, BNP, and pistons is elaborated upon a previous work,^54^ we provide a concise summary below for completeness. For ease of reference, Table 1 systematically lists important parameters and their respective value(s) we investigated or employed.

**Table 1:** Parameters and Their Value(s) Used in This Work

parameter	value(s)
solid density	7.5 m/σ^3^
fluid density	6.544 m/σ^3^
right piston pressure, *P*_1_	23, 31, 38 ε/σ^3^
left piston pressure, *P*_2_	8 ε/σ^3^
colloid volume fraction, *d*	0.01, 0.10, 0.30
pressure difference, Δ*P*	15, 23, 30 ε/σ^3^
Colloid–BNP interaction, *E*_c–p_	*E*_c–p,1_, *E*_c–p,2_, *E*_c–p,3_
colloid–colloid interaction, *E*_c–c_	*E*_c–c,1_, *E*_c–c,2_, *E*_c–c,3_

First, to construct the system, we generate the solid components, BNP and pistons, from parent bulk systems comprising DPD particles at a density of 7.5 m/σ^3^ in an initially face-centered cubic (fcc) structure. The size of the parent bulk system is 80 × 80 × 80 σ^3^ for the BNP and 10 × 80 × 80 σ^3^ for a piston. The initial bulk systems are relaxed for 100 τ with a time step of 0.01 τ to reach a thermodynamic equilibrium characterized by a steady-state value for chemical potential, temperature, and pressure. The BNP is then generated from its relaxed parent bulk by applying the method proposed by Liu and Branicio.^85^ The wavenumber is chosen to be |*q*| = 0.1 π for the cubic bulk system with edge length *L* = 80 σ, and the number of initial waves is 3000. The threshold defining the porosity level is set as *f*(*r*) < 0.385, which results in the BNP with a porosity of 65%. Alongside porosity, previous research has extensively characterized other salient features such as tortuosity, ligament size, and the pore size distribution of similar structures.^54,85^ The resultant BNP template is shown in Figure 1c. Such structure ensures continuity in both the solid and porous phases and is morphologically consistent to realistic porous structures generated by chemical treatment of spinodal decomposed glasses and metallic alloys.^86−90^ The bicontinuous structure provides a stochastic description of the pore size distribution and tortuous flow paths, which are similar to the features of natural nanoporous tight rock formations.^91−93^ We use the same BNP structure with an average pore size of 16 σ across all the simulations and focus on the influence of other important factors for colloid transport. This choice of a relatively small pore-to-colloid size ratio is designed to closely replicate the conditions within fine-grained geological formations like low-permeability shale, sandstone, and carbonate rocks, which are known for their nanoporous structures with pore sizes typically under 50 nm.^33^ Expecting a smaller pore-to-colloid size ratio in these scenarios, this setup allows us to explore the unique properties and behaviors of fluids and colloids in such nanoporous media, where conventional modeling approaches might fall short due to significant boundary layer effects.^36^ Particles in the BNP medium are immobile during the following simulations. Pistons are treated as independent rigid bodies. The total force and torque acting on each piston is calculated as the summation of the individual forces and torques applied to its constituent particles.

After assembling the BNP and two pistons, we fill the empty space with fluid particles at a density of 6.544 m/σ^3^ in an fcc structure. The fluid density is set to be slightly lower than the solid phase density to prevent fluid particles from penetrating into the solid phase. The composite system is then relaxed for 500 τ with a time step of 0.01 τ, while the periodic boundary condition is applied along *y* and *z* directions. During the relaxation, the left piston is fixed, and the right piston can move freely to reach an equilibrium position. The nanovoids are filled up through relaxation.

As a next step, we pressurize the source region in order to generate a steady pressure gradient and reduce the significant pressure fluctuation for future colloid flow. To achieve the pressurization, we set up a momentum mirror at the middle of the BNP along *x* direction. If a particle crosses the momentum mirror from the right side on a time step by a certain distance, it is reflected back to the right side by the same distance and its velocity sign along *x* direction is flipped. Then, we gradually increase the pressure applied on the right piston, while keeping everything at the left side of the momentum mirror fixed. The pressure is generated by applying specific force on each particle in the right piston. To investigate the effect of pressure and implement different pressure gradients, we apply linear force ramp from 0 to 0.3, 0.4, and 0.5 ε/σ within 250 τ simulation time with a time step of 0.005 τ on each particle in the right piston. These three force magnitudes create pressures of *P*_1_ = 23, 31, and 38 ε/σ^3^ on the right piston, respectively. The source region achieves a homogeneous pressure at the end of the pressurization process.

Subsequently, colloid particles are randomly positioned within the source region with specific volume fraction *d*. *d* is defined as the ratio of the total volume occupied by colloids to the volume of the source region by considering colloids as hard-sphere particles with a radius of 2.5 σ. To examine the effect of *d* on the colloid transport process, we consider three distinct values, as listed in Table 1, i.e., *d* = 0.01, 0.10, and 0.30. Due to variations in the source region volume under different pressures, the number of colloids placed for the same *d* varies slightly across different pressure levels. For systems with *P*_1_ = 38 ε/σ^3^ pressure in the source region, we place 86, 791, and 2387 colloids for *d* = 0.01, 0.10, and 0.30, respectively. To prevent any overlap between colloid and fluid particles, we eliminate any fluid particles whose distance of separation is within 2.5 σ from a colloid center. Then, we remove the momentum mirror and keep the maximum forces applied on the right piston from the previous step. Simultaneously, we add a force of −0.1 ε/σ on each particle on the left piston corresponding to a pressure value of *P*_2_ = 8 ε/σ^3^. This allows the colloid flow though the nanoporous media. The resultant steady pressure differences are listed in Table 1, i.e., Δ*P* = 15, 23, and 30 ε/σ^3^. During the colloid flow process, we use a constant time step of 0.005 τ. The simulation stops when the distance between the rightmost part of the BNP and the leftmost part of the right piston is smaller than 5 σ. We use the standard velocity-Verlet algorithm to update the position and velocity for each particle at each time step in all the simulations. A maximum displacement distance of 0.1 σ per time step is applied for the colloid particles to avoid the initial overlapping. We use LAMMPS^94^ with necessary packages^95−97^ to conduct all the simulations of colloid transport and OVITO^98^ to perform visualizations. As a reference, we provide the conversion between the reduced units used in our simulations and real-world in Table 2 by considering water as the reference fluid phase. However, it is important to note that this conversion is not unique; the reduced units can be scaled to match the properties of other substances, offering flexibility in interpreting our results in various physical contexts.

**Table 2:** Conversion between DPD Units and SI Units by Considering DPD Fluid Particles as Clusters of Water Molecules

quantity	DPD unit	SI unit
length	1 σ	1 nm
mass	1 M	1.50 × 10^–25^ kg
energy	1 ε	4.18 × 10^–21^ J
time	1 τ	5.99 ps
density	6.544 m/σ^3^	997 kg/m^3^
pressure	1 ε/σ^3^	4.18 mPa

To quantify the colloid transport behavior, we use the BTCs for colloids and the squared displacement for each colloid after entering the nanoporous structure. BTC offers an integrated and averaged behavior of the colloid particles. Traditionally, BTCs are determined by measuring the colloid concentration at the system outlet. Since we have all the information for each colloid, we are able to directly acquire the arrival time of colloids at different locations. Assume BNP has a length of *L* along the *x* direction, we choose four different locations inside the BNP media, 25% *L*, 50% *L*, 75% *L*, and 100% *L*, where 100% *L* is the outlet of the BNP. BTCs are plotted as the distribution of the elapsed time taken by individual colloids to travel from the BNP inlet, 0% *L*, to those locations. The elapsed time is also known as the residence time at 100% *L*. We further normalize the distribution by the total number of colloids breakthrough certain locations to avoid the influence of the number of colloids. As BTCs do not provide a distinct insight into the contribution of individual colloids and their internal localized behaviors,^27^ the analysis of squared displacement for each colloid helps bridge this gap. The squared displacement for each colloid is calculated as
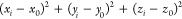
15where *x*_*i*_, *y*_*i*_, and *z*_*i*_ are the position coordinates at the current time step and *x*_0_, *y*_0_, and *z*_0_ are the position coordinates when colloids just enter the BNP media. We only count the residence time for each colloid after it enters the BNP media and stop tracking it once it is outside the BNP. Therefore, we only focus on the colloid behavior inside the nanoporous structure.

## Results and Discussion

**Figure 2:**
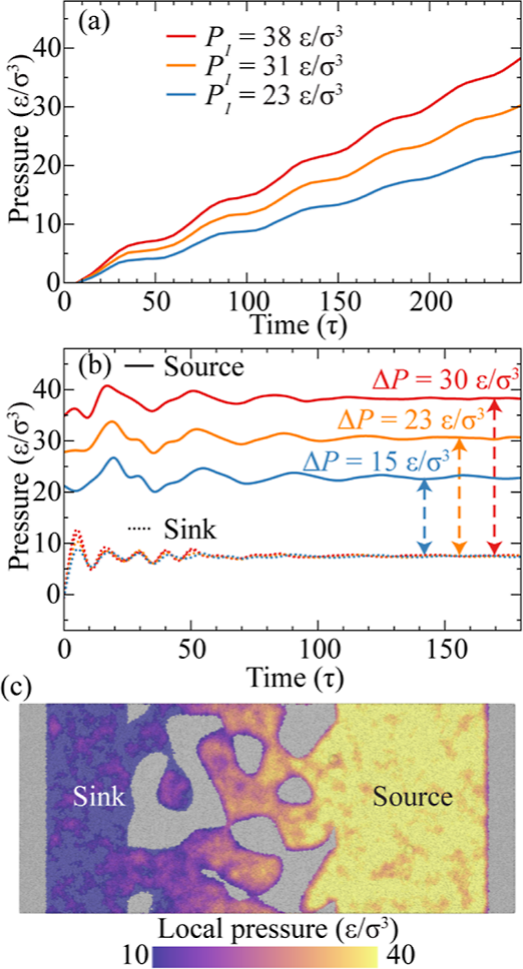
Pressure evolution in the source and sink regions for the model with *d* = 0.10, *E*_c–p_ = *E*_c–p,2_, and *E*_c–c_ = *E*_c–c,2_. (a) Average pressure in the source region during the pressurization phase. *P*_1_ is the maximum pressure level applied to the right piston. (b) Average pressure in the source and sink regions under different pressure differences during the colloid transport process. (c) Local pressure distribution for Δ*P* = 30 ε/σ^3^ at 100 τ simulation time.

In order to investigate the transport and retention of colloid within nanoporous media, we exert varying pressure levels on the right piston while maintaining a lower pressure level on the left piston. This procedure establishes a pressure differential between the source and sink regions, creating a distinct linear pressure gradient within the nanoporous structure, driving fluid and colloid flow. Directly applying pressure to the system following fluid particle relaxation can introduce substantial pressure oscillations that require an extended time to dissipate.^53^ To mitigate the effects of these oscillations, we initially pressurize the source region to achieve homogeneous pressure, as detailed in the Methods Section. Figure 2a shows the evolution of source region pressure during pressurization. This stage, occurring prior to the introduction of colloid particles, lasts for 250 τ. Here, we only consider the pressure of the fluid phase. The average pressure is derived by averaging the hydrostatic pressure of each fluid particle in the specified region based on the virial stress. As evident in Figure 2a, even though the pressure on the right piston increases linearly, the pressure in the source region shows a gradual rise interspersed with fluctuations due to the inherent dynamics of the simulation environment. This phenomenon reflects the complex interplay of forces and is indicative of the adaptive response within the simulated system as it seeks equilibrium.

Figure 2b presents the average pressures in both the source and sink regions as a function of simulation time during the colloid transport process, i.e., following colloid introduction and subsequent release of the momentum mirror. Although pressure oscillations are evident in the source region, their amplitudes are considerably milder than those observed in simulations without prior pressurization.^54^ These oscillations gradually dissipate as the simulation progresses. Steady pressures in the source regions are achieved after 100 τ and in the sink regions after 60 τ. At the steady state, the source region exhibits pressure levels of 23, 31, and 38 ε/σ^3^, as a result of the pressures exerted on the right piston. A similar observation is made in the sink region, where the equilibrium pressure registers at 8 ε/σ^3^. The pressure differential between the source and sink regions, denoted as Δ*P*, aligns with the difference between pressures on the right and left pistons, *P*_1_ – *P*_2_, i.e., Δ*P* = 15, 23, and 30 ε/σ^3^. We further determine the local pressure at each fluid particle position by averaging the hydrostatic pressure within a 3 σ radius from its center. Figure 2c displays the local pressure distribution of one simulation model under Δ*P* = 30 ε/σ^3^ in the steady pressure state. Despite inherent thermodynamic fluctuations, the local pressures in the source and sink regions show discernible values, while the local pressure within the nanoporous media exhibits a continuous decline from the source to the sink region.

**Figure 3:**
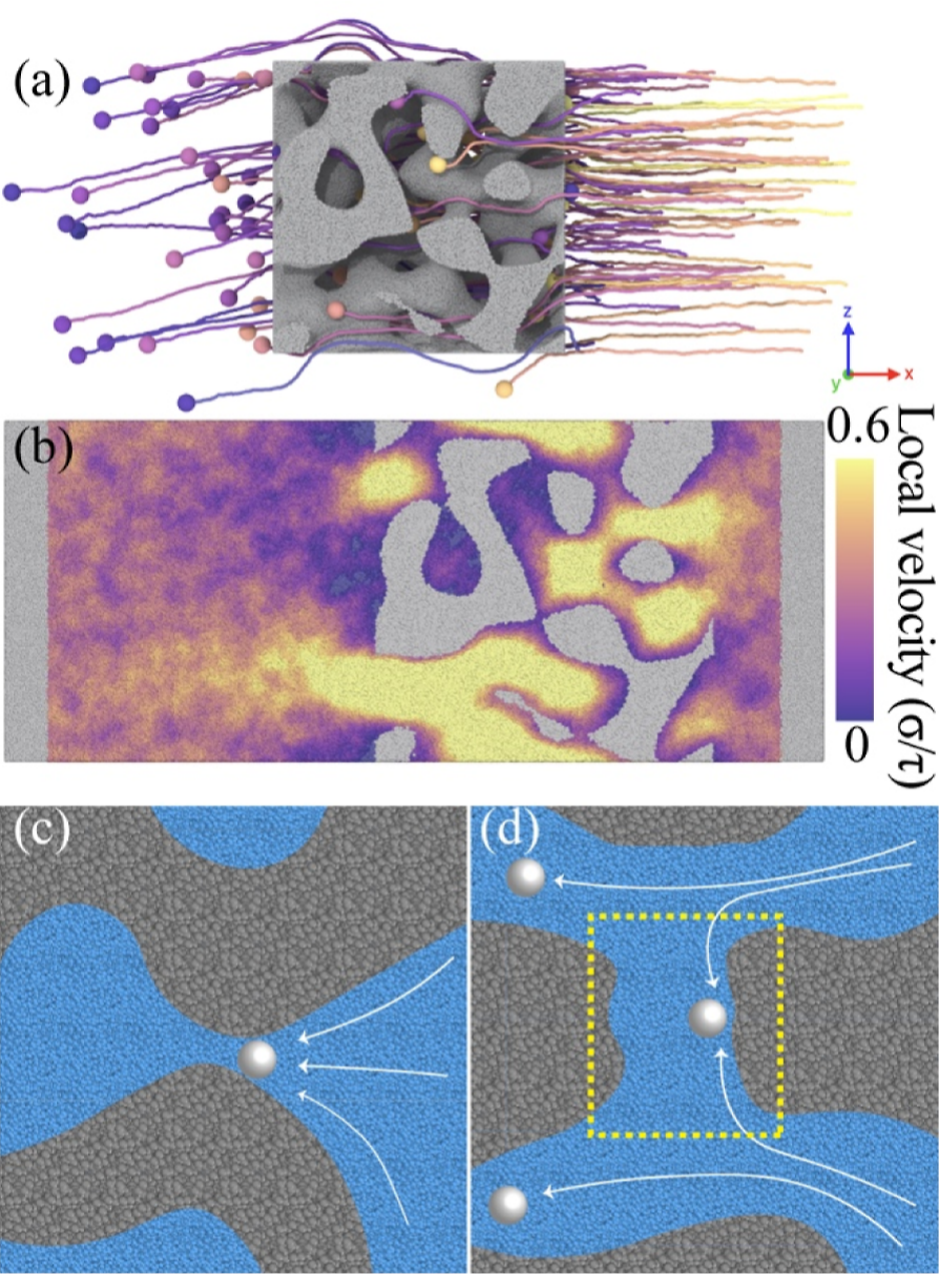
Colloid transport processes. (a) Visualization of colloid trajectories during the simulation time for the model with *d* = 0.01, *E*_c–p_ = *E*_c–p,2_, *E*_c–c_ = *E*_c–c,2_, and Δ*P* = 30 ε/σ^3^. Colloids and their corresponding trajectories are colored by their initial positions, as shown in Figure 1b. Trajectories are unwrapped at periodic boundaries to make the particle trajectories continuous. (b) Local fluid velocity distribution during colloid transport. Two colloid retention mechanisms observed under unfavorable attachment condition (*E*_c–p,1_) are shown in (c,d). (c) Straining and (d) trapping in low-flow zone. Gray and blue regions represent porous media and fluid, respectively. White sphere particles represent colloids. White arrows indicate possible colloid trajectories. Yellow dotted square highlights low flow zone.

To elucidate the transport and retention mechanisms of colloids, we use a particle-based model, which provide the complete state and trajectories of the colloids. A representation of the model with parameters *d* = 0.01, *E*_c–p_ = *E*_c–p,2_, *E*_c–c_ = *E*_c–c,2_, and Δ*P* = 30 ε/σ^3^ is illustrated in Figure 3a. For clarity, we removed the fluid and piston particles, focusing only on the trajectories of the colloids and their interactions with the nanoporous structure. We adjusted the trajectory lines at the periodic boundaries, namely, *y* and *z*, to ensure continuity. An animation showing colloid transport trajectories is provided in the Supporting Information as Movie S1. In Figure 3a, it is evident that colloids move linearly along the *x*-axis with minor fluctuations until they enter the nanoporous structure. Within the nanostructure, their trajectories become complex due to physical obstruction and interactions between the colloids and BNP. Upon exiting the BNP structure, the colloids resume linear movement based on their exit flow direction, suggesting a significant influence of the BNP structure on colloid flow. By the end of the simulation, when the right piston approaches the BNP right side, several colloids remain within the porous structure. One should note that some colloids remain in this structure because of insufficient fluid flow, especially those initially near the right piston, such as the yellow-colored particles shown in Figure 3a. These colloids could potentially leave the BNP structure with an influx of additional fluid. However, we still observe the retention of colloids characterized by their persistent stagnation within the BNP for a long simulation time. The local velocity distribution along the *x*-axis for this system is displayed in Figure 3b. When determining the local velocity, the *x* component of the velocity is averaged across each particle and its surrounding particles within a 3 σ cutoff. It is evident that the flow within the nanoporous media is inhomogeneous, with certain preferred channels exhibiting significantly higher velocity than others. These faster channels for fluid flow also act as the primary routes for colloid transport.

Through a close examination of the colloid trajectories, we can discern the mechanisms responsible for colloid retention in the stochastic BNP structure. Beyond the simple attachment of colloids to the BNP surface owing to colloid–BNP attraction under favorable attachment conditions, it is important to study retention mechanisms under unfavorable attachment conditions characterized by significant colloid–BNP repulsion. These distinct conditions are achieved by modulating the energy well depth of *E*_c–p_, as shown in Figure 1a. Here, we consider *E*_c–p,1_ as an unfavorable attachment condition where the colloid–BNP attraction is negligible. Under the unfavorable attachment condition, we are able to isolate the effect of interfacial interactions and gain insights into colloid retention influenced by alternative mechanisms. Two primary retention mechanisms under these unfavorable attachment conditions observed in the simulations are straining and trapping in low-flow zone, as illustrated in Figure 3c,d. Straining occurs when colloids become physically trapped within pore throats narrower than their size. Low-flow zones, alternatively termed as immobile zones, represent areas where local flow is either sluggish or stagnant. This phenomenon is commonly observed in fluid flow through intricate nanoporous structures, as visualized by the purple regions in Figure 3b. While the majority of colloid trajectories align with the primary fluid stream during transport, some deviate due to interactions with the media surface or other colloids, subsequently becoming entrapped in low-flow zones. The stationary nature of these zones makes it challenging for such colloids to extricate themselves. Movies S2 and S3 in the Supporting Information show an example of each retention mechanism discussed above.

**Figure 4:**
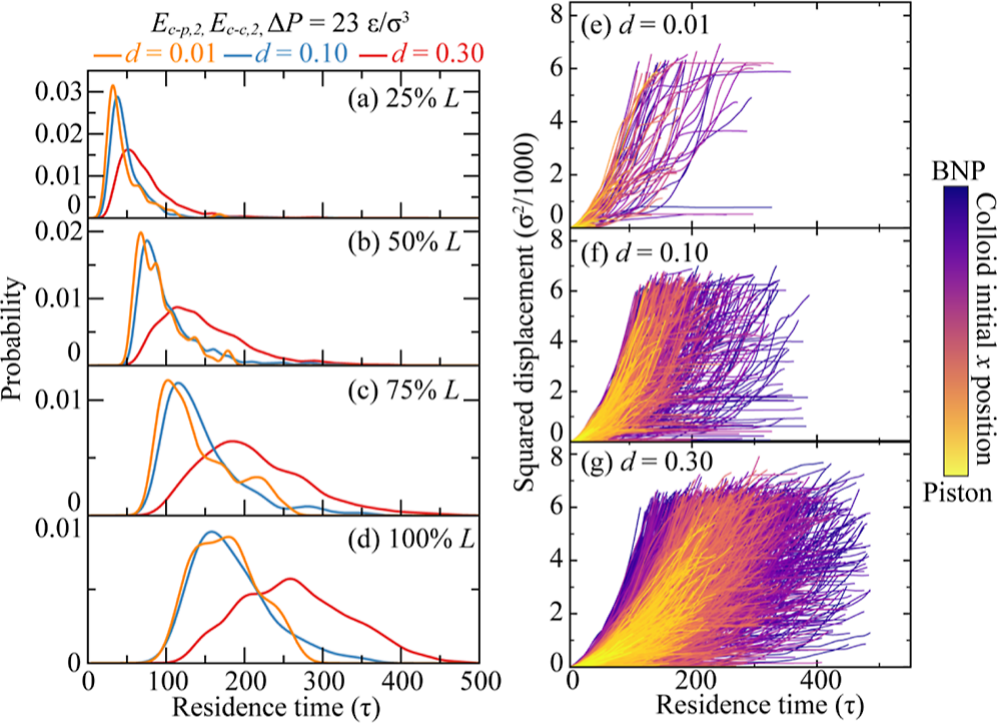
Colloid volume fraction effect on colloid transport in BNP media. (a–d) Breakthrough curves when colloids breakthrough 25–100% *L* of the BNP media. Different colors represent different colloid volume fraction, *d* = 0.01–0.30, while *E*_c–p_ = *E*_c–p,2_, *E*_c–c_ = *E*_c–c,2_, and Δ*P* = 23 ε/σ^3^. (e–g) Squared displacement of each colloid for *d* = 0.01, 0.10, and 0.30 as a function of residence time. Curves are colored by colloids’ initial positions along the *x* direction.

We further examine the influence of different factors on colloid transport in nanoporous media. We start by focusing on the effect of colloid volume fraction, denoted as *d*. The BTCs for models with *d* = 0.01, 0.10, and 0.30 measured at different locations are shown in Figure 4a–d. The other three parameters, *E*_c–p_, *E*_c–c_, and Δ*P*, are kept at intermediate values. By analyzing the BTCs across locations, we extract statistical data on colloid transport within the BNP media. One consistent trend for different *d* values is the widening of the BTCs as colloids penetrate further into the nanoporous framework. This is due to cumulative interactions between the colloid and the surface of the BNP media. All BTCs exhibit a consistent pattern: a slightly rightward-skewed distribution, with diminishing skewness as the colloids penetrate deeper into the media. The observed early breakthrough and extended tailing, as depicted in Figure 4a–d, are indicative of anomalous colloid transport. This deviation from typical symmetric diffusion behavior is similar to phenomena previously described by Fan et al.^99^ For the smallest volume fraction, *d* = 0.01, where only 86 colloids are considered, the BTCs are less distinct compared to those with higher *d* values. However, the impact of *d* on the integrated colloid transport is evident. At lower *d* values, specifically *d* = 0.01 and 0.10, the BTCs are similar, indicating minimal colloid–colloid interactions and an insufficient colloid count to saturate the BNP media. However, as *d* increases to 0.30, BTC peaks shift to longer times and become broader relative to lower *d* values, indicating a more extended average breakthrough time at each location, which is evidence of increased anomalous transport behavior. This shift arises because the interaction of the BNP structure with colloids is increased, while it accommodates such a substantial colloid volume fraction. It is important to note that the BTCs are normalized; thus, even if the BTC height is diminished for *d* = 0.30, the actual colloid count in the system might be significantly high. A substantial colloid volume fraction can lead to clogging due to colloid interaction with the BNP structure and an increased chance of interactions between colloids, both of which contribute to the sluggish transport behavior observed.

A deeper insight can be gained by evaluating the displacement of individual colloids. Given the complex porous architecture, the maximum squared displacement varies based on the trajectories followed during colloid transport. As shown in Figure 4e–g, multiple events may occur during the colloid transport process, including continuous colloid flow, colloid retention, and colloid remobilization. When contrasted with the model with *d* = 0.01, the model with *d* = 0.30 exhibits a notably wider spectrum of residence times. This observation agrees with the BTC outcomes, which indicate an increase in anomalous transport occurrences for larger *d* values. Furthermore, the results show that the initial position of the colloid, specifically its proximity to the BNP structure, has a discernible effect on its transport behavior. As demonstrated in Figure 4g, colloids that begin their journey further away from the BNP (represented in yellow) showcase a more constrained range of displacement and residence time than those that start closer to the BNP (illustrated in purple). This suggests that the quantity of colloids already present within the media can influence the transport behavior of incoming colloids. This is primarily due to pre-existing occupation of sites, which subsequently restricts additional colloid–BNP interactions and retention.

**Figure 5:**
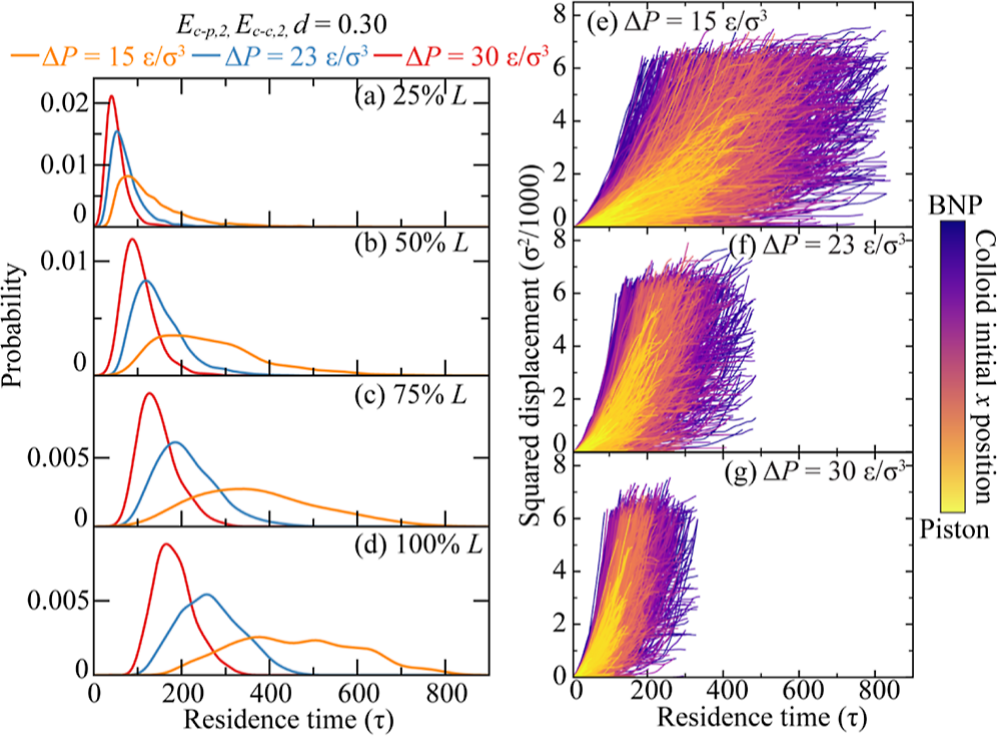
Pressure effect on colloid transport in BNP media. (a–d) Breakthrough curves when colloids breakthrough 25–100% *L* of the BNP media. Different colors represent different pressure differences, Δ*P* = 15–30 ε/σ^3^, while *E*_c–p_ = *E*_c–p,2_, *E*_c–c_ = *E*_c–c,2_, and *d* = 0.30. (e–g) Squared displacement of each colloid at Δ*P* = 15, 23, and 30 ε/σ^3^ as a function of residence time. Curves are colored by colloids’ initial positions along the *x* direction.

We perform similar analyses to explore the effects of other factors on colloid transport in the BNP media, including pressure difference Δ*P*, colloid–BNP interaction, *E*_c–p_, and colloid–colloid interaction, *E*_c–c_. For Δ*P*, we maintain *E*_c–p_ and *E*_c–c_ at intermediate strengths and use a large *d* = 0.30 for enhanced statistical representation. BTCs for models subjected to varying pressures are shown in Figure 5a–d. These findings indicate a significant effect of Δ*P* on colloid transport dynamics: elevated Δ*P* values enhance colloid transport velocity and reduce the colloid retention rate. Furthermore, BTCs at higher Δ*P* values display less skewness compared to those at lower Δ*P*, suggesting that increased Δ*P* values diminish the anomalous diffusion of colloids. This Δ*P* effect is also present in the colloid displacement curves shown in Figure 5e–g. A distinct observation is the reduced colloid residence time at Δ*P* = 30 ε/σ^3^ relative to Δ*P* = 15 ε/σ^3^. Additionally, Figure 5e shows a higher number of colloids retained by the BNP, as evidenced by a predominant occurrence of flat displacement curves for extended residence times. For further reference and analysis, we include Figure S1 in the Supporting Information. This figure utilizes the data from Figure 5, with the *x*-axis representing the product of residence time and Δ*P*, offering an additional perspective on the results.

**Figure 6:**
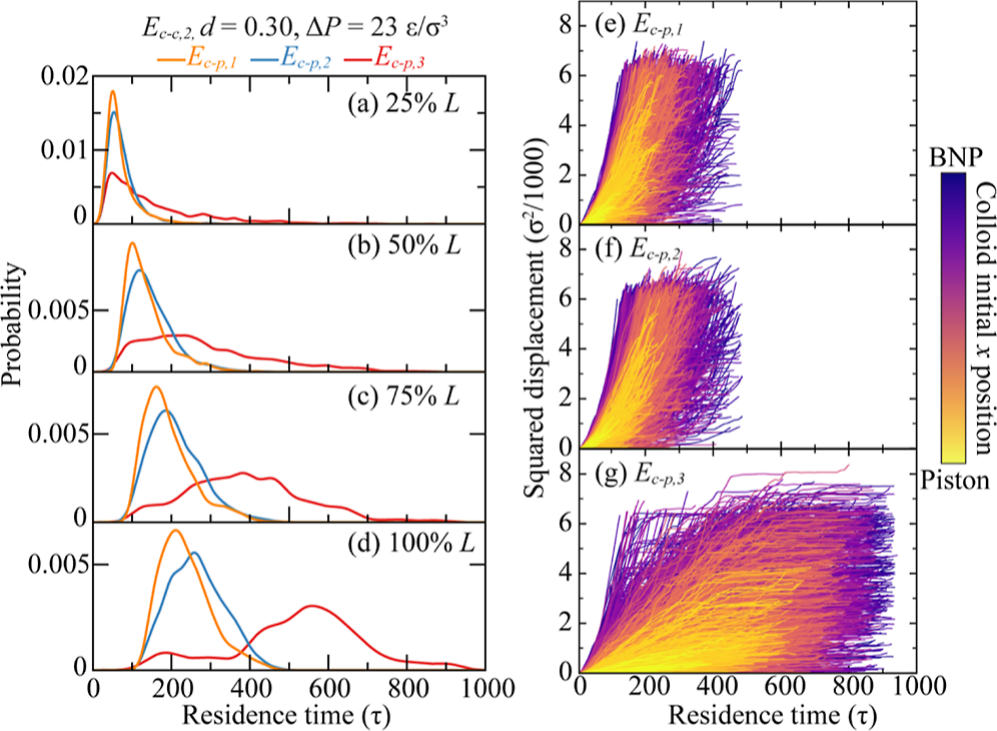
Colloid–BNP interaction effect on colloid transport in BNP media. (a–d) Breakthrough curves when colloids breakthrough 25–100% *L* of the BNP media. Different colors represent different *E*_c–p_, *E*_c–p_ = *E*_c–p,1_ to *E*_c–p,3_, while *E*_c–c_ = *E*_c–c,2_, *d* = 0.30, and Δ*P* = 23 ε/σ^3^. (e–g) Squared displacement of each colloid at *E*_c–p_ = *E*_c–p,1_, *E*_c–p,2_, and *E*_c–p,3_ as a function of residence time. Curves are colored by colloids’ initial positions along the *x* direction.

Figure 6 illustrates the effects of *E*_c–p_, with both Δ*P* and *E*_c–c_ held at intermediate levels and *d* = 0.30. We modulate the interaction strengths between colloids and the BNP to investigate the effect of boundary layer on colloid transport. Given the nanoscale size of the channels and the small ratio of pore size to colloid size, the boundary layer effect is indeed significant. The associated BTCs are shown in Figure 6a–d. At 25% *L*, a location close to the BNP inlet, the BTCs for varying *E*_c–p_ interactions all peak around the same residence time. However, as shown in Figure 6d, when colloids penetrate deeper into the BNP media, the peak locations begin to diverge. Notably, stronger *E*_c–p_ attractions result in peaks shifting to longer residence times compared to those with weaker *E*_c–p_ attractions. This trend highlights the cumulative impact of colloids engaging with the collector surface, particularly where the stronger colloid–BNP forces slow down the progression of colloids through the media more significantly as they travel further. Thus, the influence of *E*_c–p_ becomes increasingly prominent at deeper penetrations. Additionally, the BTC amplitude diminishes for increasing *E*_c–p_ attraction intensity, indicating that heightened *E*_c–p_ attractions result in greater colloid retention. This trend highlights the significant contribution of the boundary layer to the control of colloid transport within nanoporous structures.

As shown in Figure 6e–g, the trajectories of individual colloids under varying *E*_c–p_ interactions differ considerably. Beyond the notably elongated residence times at *E*_c–p,3_, a considerable fraction of colloids remains trapped within the nanoporous structure as the predominant flatness of displacement curves in the latter simulation phases attests. The initial positioning of colloids also affects their transport under *E*_c–p,3_. Colloids located further from the BNP media demonstrate reduced displacement magnitudes, as shown in Figure 6g. This phenomenon arises from the pronounced colloid retention within the nanoporous structure early on, which subsequently causes clogging and jamming that constrain the movement of later-arriving colloids.

**Figure 7:**
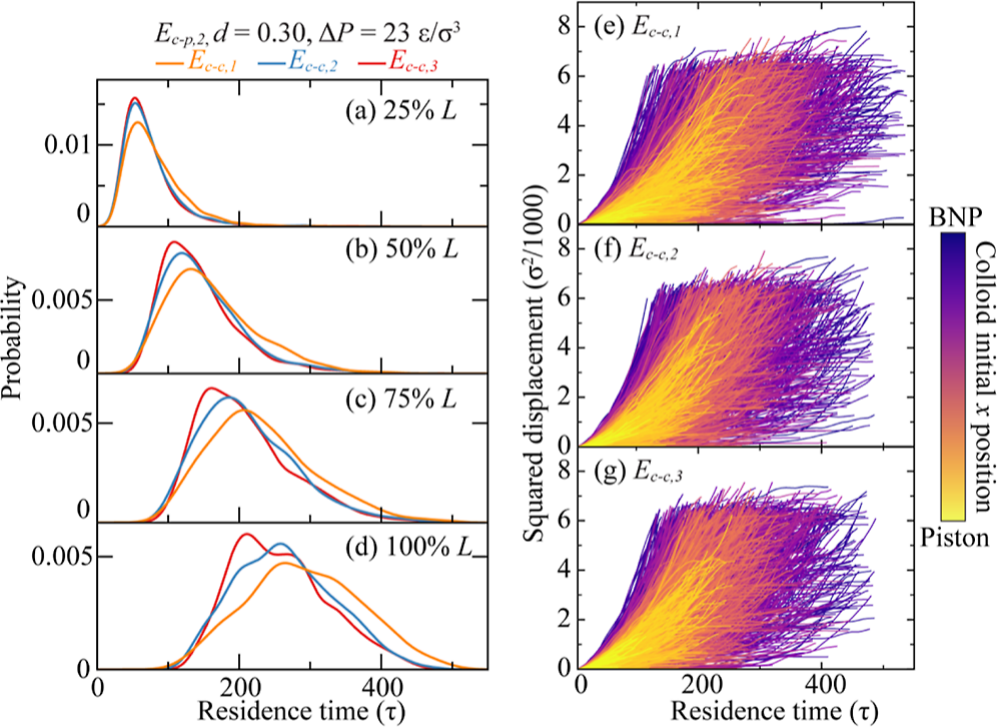
Colloid–colloid interaction effect on colloid transport in BNP media. (a–d) Breakthrough curves when colloids breakthrough 25–100% *L* of the BNP media. Different colors represent different *E*_c–c_, *E*_c–c_ = *E*_c–c,1_ to *E*_c–c,3_, while *E*_c–p_ = *E*_c–p,2_, *d* = 0.30, and Δ*P* = 23 ε/σ^3^. (e–g) Squared displacement of each colloid at *E*_c–c_ = *E*_c–c,1_, *E*_c–c,2_, and *E*_c–c,3_ as a function of residence time. Curves are colored by colloids’ initial positions along the *x* direction.

The last factor we investigate is the colloid–colloid interaction intensity, *E*_c–c_. Similar to previous cases, we maintain Δ*P* and *E*_c–p_ at intermediate levels, while setting *d* at its maximum value of 0.30. The results are presented in Figure 7. As evident from the BTCs in Figure 7a–d, an increase in *E*_c–c_ attraction promotes faster colloid transport and diminishes colloid retention. In situations of pronounced repulsion between colloids, the colloid–colloid interactions within the BNP channel can cause colloids to deviate from the channel center path. Here, the reference to a repulsive force is related to the behavior of the colloids when they come within a certain proximity where the interaction potential repulsive component predominates—specifically when the potential energy gradient between the colloids becomes negative due to a decrease in interparticle distance, as shown in Figure 1a. The deviation from the channel center path increases the probability of colloids engaging with the collector surface, subsequently constraining colloid transport and increasing the retention rate. This trend becomes more pronounced deeper within the channel, as demonstrated by the growing divergences among BTCs at 100% *L*. Nonetheless, the overall differences in BTCs across various *E*_c–c_ values are less pronounced compared to other factors. This can be attributed to the nanoscale channels incapacity to house concurrently a substantial quantity of colloids, thus restricting the extent of colloid–colloid interactions within the BNP channel. The squared displacement curves for individual colloid with varying *E*_c–c_ values are shown in Figure 7e–g. Similar to the observations in BTCs, colloids with *E*_c–c,1_ exhibit a marginally broader residence time distribution compared to those with *E*_c–c,3_, even though their overarching behaviors remain analogous.

**Figure 8:**
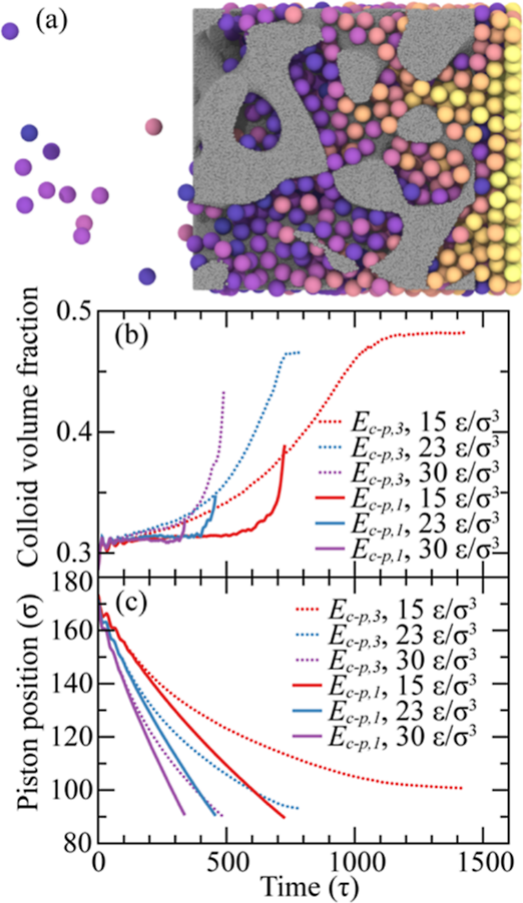
Colloid jamming process during colloid flow through BNP media. (a) Colloid jamming for *E*_c–p_ = *E*_c–p,3_, *E*_c–c_ = *E*_c–c,2_, *d* = 0.30, and Δ*P* = 15 ε/σ^3^. Colloids’ color coding follow the scheme used in Figure 1. (b) Colloid volume fraction evolution in the source region. (c) Right piston position for different samples during the simulation time.

In our simulations of colloid transport across various conditions, we identify instances of colloid jamming in the proximity of the BNP media inlet, especially under strong *E*_c–p_ attractions, high *d*, and small Δ*P* values. The influence of *E*_c–c_ on jamming is relatively minimal in comparison to other factors due to the limited nanoporous channel capacity and absence of colloid aggregation. Interestingly, while colloid–colloid interaction did not significantly contribute to jamming, we noted a reduced colloid retention rate with increasing colloid–colloid attraction strength, which in turn slightly mitigated jamming within the BNP channels. Such jamming is characterized by a halt in colloid movement and sluggish advance of the pistons. An illustration of colloid jamming is shown in Figure 8a, where the BNP media are saturated and unable to accommodate additional colloids. Consequently, incoming colloids cannot infiltrate the BNP structure and instead accumulate and compact in the source area. This leads to the formation of compact layers akin to crystal structures outside the nanoporous media. Such jamming phenomenon can primarily be ascribed to the characteristics of the nanoscale channel, specifically the significant ratio between colloid and channel dimensions, coupled with pronounced boundary layer effects. In addition, the nondeformable nature of the colloids in our simulations can affect their trajectories, particularly in relation to jamming phenomena. Such rigid colloids, when subjected to narrow pore spaces and pressure, are prone to form dense assemblies that can act as bottlenecks and further accentuate the likelihood of jamming.

To gain further insights into the jamming process, we also evaluate changes in the colloid volume fraction in the source region over the simulation time for models with *d* = 0.30 across diverse *E*_c–p_ and Δ*P* values, as shown in Figure 8b. The results reveal that without substantial *E*_c–p_ attraction, i.e., *E*_c–p,1_, the colloid volume fraction in the source region remains approximately at 0.30, until near the end of the simulation. This consistent volume fraction points to a steady colloid transport process. The marked uptick toward the end of the simulation is linked to a single layer of colloids that, being physically attached externally to the media, remain stationary for the simulation duration. As the right piston approaches the BNP and the volume of the source region contracts, these stationary colloids contribute to a swift surge in the colloid volume fraction. Therefore, this can be considered a finite size effect artifact of the simulations. In contrast, under conditions with pronounced *E*_c–p_ attraction, we observe a slow and steady increase in volume fraction in the source, indicating differential flow rates between colloids and fluid particles. Such phenomenon becomes more evident at reduced Δ*P* = 15 ε/σ^3^, where the volume fraction plateaus of 0.48 at ∼1200 τ, indicating the onset of colloid jamming.

In line with previous studies, jamming in colloidal systems is observed only when the colloid volume fraction surpasses a specific threshold, known as the jamming threshold.^100−102^ This threshold is dependent on various factors, such as the pore-to-colloid size ratio, colloid deformability, geometry of the confinement, environmental temperature, and pressure.^103,104^ In our simulations with the specific condition (*E*_c–p_ = *E*_c–p,3_, *E*_c–c_ = *E*_c–c,2_, *d* = 0.30, and Δ*P* = 15 ε/σ^3^), we identify the critical threshold for jamming to be around a colloid volume fraction of 0.48. Notably, this threshold slightly lowers when Δ*P* is increased to 23 ε/σ^3^. The occurrence of jamming at high colloid volume fractions aligns well with findings from previous studies.^105−107^ These studies suggest that dense colloid suspensions, typically a colloid volume fraction ranging from 0.2 to 0.6, are more prone to jamming when the local volume fraction reaches critical threshold.

These results are reflected in the evolution of the right piston position, as shown in Figure 8c. According to the results, while Δ*P* predominantly determines the piston velocity, the shape of the curves is modulated by *E*_c–p_. In scenarios with *E*_c–p_ = *E*_c–p,1_, the right piston progresses linearly at a consistent rate toward the BNP media. However, when *E*_c–p_ = *E*_c–p,3_, the piston velocity progressively decreases throughout the simulation. When the piston velocity nearly halts under reduced Δ*P* scenarios, the colloid jamming shown in Figure 8a occurs. Overall, these results provide comprehensive insights into colloid transport dynamics within stochastic nanoporous media.

The results presented in this study provide significant insights into the pore-scale mechanisms that dictate colloid retention under diverse physicochemical conditions. Such understanding is crucial for enhancing control over colloid transport in intricate porous media. While various colloid retention mechanisms have been suggested based on experimental colloid BTCs,^8^ characterizing the intricate dynamics of colloid transport and retention in porous media remains a challenge. Several research efforts have sought to tackle this challenge by employing techniques such as confocal microscopy to directly observe colloid transport within microsized channels.^27,108−110^ However, recent findings regarding nanoporous materials and nanoconfined fluid behavior highlight the critical need for a deeper understanding of the mechanisms governing colloid transport in complex nanoporous materials.^28,31,33,34^

Our findings suggest that under favorable attachment conditions, the primary mechanism for colloid retention stems from the attachment of colloids to the collector surface, driven predominantly by the considerable *E*_c–p_ attraction. However, other colloid retention mechanisms are independent of *E*_c–p_ and rather closely related to flow regimes and pore structures.^111^ It is insightful to reveal the colloid retention mechanisms in nanoporous media without colloid-collector attraction by taking advantage of particle dynamics models. We hypothesize that retention mechanisms linked to attractive *E*_c–p_ likely become ineffective at *E*_c–p,1_, implying that any residual colloid retention arises from noninterfacial mechanisms. By analyzing 3D colloid trajectories, we directly observe two predominant pore-scale mechanisms responsible for colloid retention within the BNP structure under less-favorable attachment conditions, as depicted in Figure 3c,d and in Movies S2 and S3. The first is straining, identified as the primary retention mechanism for colloids in porous media under unfavorable attachment conditions.^112^ The second mechanism is trapping in low-flow zone. Figure 3b demonstrates the emergence of low-flow or even reverse-flow areas due to the stochastic nature of porous topology. Colloids entering these regions become trapped without adhering to collector surfaces, a mechanism potentially more relevant for nanometer-sized colloids.^6^ The influence of low-flow or stagnation areas on colloid retention has been posited in numerous prior experimental and modeling investigations.^11,14,111,113,114^ For example, Tong and Johnson^114^ conducted a study on colloid retention in both porous media and impinging jet systems using carboxylate-modified microspheres of varying sizes under both favorable and unfavorable attachment conditions. Their results revealed that the colloid retention rate in porous media was 5–50 times higher than that in impinging jet systems. They suggested that a portion of this increased retention in porous media could be attributed to retention in stagnant flow zones, underscoring the importance of pore geometry in governing colloid retention. Therefore, our study effectively replicates the colloid transport and retention phenomena in nanoporous media, aligning closely with findings from earlier studies previously alluded to.

In this study, our focus lies on understanding how factors such as *d*, Δ*P*, *E*_c–p_, and *E*_c–c_ impact colloid transport within nanoporous media. One of the key advantages of particle dynamics model is its flexibility in parameter value selection. This adaptability facilitates the exploration of a wide range of these parameter values, mimicking various physicochemical conditions and thereby enabling an exhaustive examination of their implications. To bridge *E*_c–p_ and *E*_c–c_ with physical experiment scenarios, it is instructive to consider the role of solution chemistry, particularly ionic strength and pH. Generally, an increase in ionic strength leads to a more pronounced colloid–collector potential energy minimum, which in turn enhances the attraction between colloids and collectors.^115−117^ Similar to the colloid–collector interaction, changes in solution chemistry are known to significantly affect colloid–colloid interactions.^118,119^ For instance, Kusova et al.^118^ have shown that certain conditions, such as an intermediate pH combined with low ionic strength, promote colloid–colloid attraction. In this context, the varying *E*_c–p_ and *E*_c–c_ interactions employed in our simulations can be seen as representations of varied solution chemistry conditions.

Previous research has highlighted the significance of these factors in microporous media.^110,116,120−125^ For example, in column experiments, a decline in the retention rate was observed with an increase in *d* under intermediate *E*_c–c_ conditions. However, the influence of *d* on colloid retention proved minimal under extreme *E*_c–c_ repulsion or attraction conditions.^116^ In contrast, a large *d* was found to lead to greater retention under conditions of strong *E*_c–c_ attraction since the retained colloids acted as new retention sites for subsequent colloids in suspension.^123^ Strong *E*_c–c_ attraction can also facilitate colloid aggregation, further enhancing retention.^124^ Additionally, a larger *d* not only extends the transport distance for colloids but also affects local colloid concentrations, which in turn influences subsequent colloid transport.^121^ These findings highlight the complex interplay among various factors, emphasizing the importance of understanding their combined effects on colloid behavior. Nevertheless, the specific impacts of these factors on colloid transport through nanoporous media remain less explored.

The findings reported here highlight the effects of nanoporous media characterized by a sizable colloid-to-pore size ratio coupled with pronounced boundary layer effects on colloid transport. The three values used for each factor—two representing extreme values and one intermediate—are carefully chosen to highlight the changes in colloid transport behavior. While incorporating a more extensive data set could provide additional granularity, our analysis suggests that the inclusion of further data would likely reaffirm rather than modify the core conclusions of our study. To study size effects in our current simulation systems, we perform a simulation with both the system size and the number of colloids increased fourfold, i.e., doubling in *y* and *z* directions. The results are shown in Figure S2 in the Supporting Information. This comparative analysis reveals no significant deviations between the BTCs and colloid displacement patterns of the two systems, therefore validating our current simulation dimensions and colloid numbers as adequate for a comprehensive sampling of the phase space, supporting the ergodicity assumption.

Notably, the influence of various factors on colloid transport in nanoporous media exhibit nuances when contrasted with their effects in microporous media. Detailed evaluation of these effects, based on the analysis of BTCs and individual colloid displacement curves, is provided in Figures 4–8. In nanoporous channels, a larger value of *d* fosters colloid clogging, attributable to the restricted pore space in the BNP, thereby resulting in increased colloid retention. Conversely, higher Δ*P* contributes to faster fluid flow that results in significantly reduced colloid residence time and retention rate. A strong *E*_c–p_ attraction markedly influences colloid retention by enhancing colloid attachment to the collector surface. In extreme scenarios, this can precipitate colloid jamming, halting subsequent colloid flow.

The impact of *E*_c–c_ on colloid behavior within nanoporous media, while minimal, diverges from observations in microporous channels. In microporous environments, increased *E*_c–c_ attraction can lead to colloid aggregation, potentially heightening retention due to the formation of larger clusters and additional attachment points. However, in the context of our study, the dimensions of the nanoporous channels inherently limit the accumulation of colloids. This constraint, coupled with the relatively high pressure and enhanced flow characteristic of our simulated environment, reduces the likelihood and subsequent impact of significant colloid aggregation. Interestingly, our data suggest that an increase in colloid–colloid attraction tends to align colloids more closely with the flow direction, minimizing interactions with the media and thus reducing overall retention.^54^ On the other hand, decreased attraction or increased repulsion tends to deflect colloids toward the media walls, promoting retention. This nuanced behavior underscores the distinct dynamics of colloid transport in nanoporous media compared to microporous systems.

Furthermore, the initial position of the colloid during the flow also plays a part in determining its behavior. As suggested by the broader distributions of displacement and residence time in squared displacement curves, colloids introduced into the BNP earlier often exhibit more anomalous behavior than those entering later. In essence, the findings furnish a lucid comprehension of how various factors modulate colloid transport and retention in complex nanoporous media.

The literature highlights additional factors that influence colloid transport in porous media, such as colloid size^110,114,126^ and collector surface roughness.^127−130^ In the simulations described here, colloids are modeled as hard-sphere-like particles, with a deliberated substantial colloid-to-pore size ratio, reflecting a nanoporous environment. Varied colloid-to-pore size ratios can be attained by adjusting the size of the nanoporous structure or by modulating colloidal interactions. It is pertinent to highlight that the nanoporous structure used in this study, namely, the BNP structure, features well-defined topological attributes and a seamless surface devoid of grain-to-grain contacts. Although the BNP structure is formed by discrete particles, these particles are densely packed and overlap, resulting in a surface that is effectively smooth with negligible roughness. Therefore, the effect of collector surface roughness on colloid transport is excluded from our simulations. Moreover, the hydrophobicity of colloids is known to significantly impact colloid transport.^131^ Prior studies have shown that varying the attraction strength within the DPD model can effectively mimic different levels of hydrophilicity.^61,132,133^ Within the scope of our simulations, we maintain a constant fluid interaction parameter, representing a constant hydrophobicity across different simulations, to focus on the impacts of colloid–colloid and colloid–porous media interactions. Additionally, all simulations are conducted for colloid transport in saturated porous media. It is worth mentioning that the colloid transport and retention mechanisms, as well as the effects of various factors, may differ in the context of unsaturated porous media.^110,123,134^

This work demonstrates the effective application of particle dynamics models to simulate colloid transport through stochastic nanoporous media, building upon prior research that utilized similar approaches for modeling colloid suspension rheology and transport within idealized geometries, such as those based on Happel’s cell.^50,51,59,60,77,78^ Notably, this study establishes a comprehensive framework for simulating colloid transport under diverse conditions, leveraging previous fluid dynamics research within nanoporous environments.^54^ This allows for an extensive parametric analysis over a broad spectrum of physicochemical conditions, offering a robust foundational understanding that will inform future theoretical and empirical research into colloid dynamics in highly specific contexts.

Furthermore, our simulation approach provides the capability to trace individual colloid trajectories, shedding light on pore-scale interactions under conditions traditionally challenging to analyze through both mechanistic and continuum modeling techniques, as well as experimental approaches. This capability is especially valuable for examining detailed colloid behaviors, such as squared displacement under varying conditions. Complementing the BTCs, the displacement data of individual colloids provides insights into the unique journey of each colloid, offering insights beyond bulk transport behaviors. This level of detail enables us to discern specific behaviors such as retention events and subsequent mobilization on a microscale, which is critical for understanding the heterogeneity of transport in porous media. Moreover, tracking individual colloids enables the examination of the effect of colloid starting positions, which is a fundamental variable that can greatly affect their eventual pathways and interactions within the nanoporous structure. By examining these individual paths, we can analyze how proximity to the BNP interface influences the likelihood of colloid retention and remobilization, thereby providing a better understanding for colloid dynamics. The distinct position of colloids also reflects real-world scenarios, where initial placement significantly dictates subsequent transport dynamics, further enhancing the relevance and applicability of our findings.

Additionally, we emphasize the particular relevance of our work to the study of colloid transport in nanoporous structures, where nanoconfinement and boundary layer effects play a crucial role. As discussed in the previous section, our results uncover subtle yet significant differences in how various factors impact colloid transport in nanoporous media, as opposed to their effects in microporous media. These findings offer novel insights and contribute to a more nuanced understanding of colloid dynamics, addressing gaps not extensively explored in the existing literature.

We recognize the importance of acknowledging potential limitations in our study and identifying future research paths. In order to ensure the validity of our findings within the context of our study scope, we design our simulation environment carefully to balance the need for statistical robustness with computational efficiency. Despite the limited number of pores and flow channels, the domain is constructed to be representative enough to capture the critical dynamics of colloid transport within nanoporous media. While extending the domain could offer additional insights, we expect that such an increase in domain size would unlikely alter the fundamental behaviors and conclusions presented in this study based on our extensive simulations and analysis.

Besides, based on our current simulation results, we do not observe apparent colloid aggregation, even with the strongest colloid–colloid interaction *E*_c–c,3_. This absence of aggregation could be due to the relatively high-pressure gradient applied, which may have counteracted the aggregative forces. The dynamics of colloid aggregation are complex and depend on a balance of forces, including attraction between colloids and the hydrodynamic forces imposed by the fluid flow. It is plausible that the flow conditions in our simulations are sufficient to prevent the formation of larger aggregates despite the strong colloid–colloid attraction. The in-depth exploration of how colloid–colloid interactions influence the behavior of colloid suspensions under a variety of conditions remains a critical area for future research. Moreover, in order to add more realism, future modeling efforts could incorporate the deformability of colloids and more complex features of the nanoporous matrix.

## Conclusions

In this study, particle dynamics models served as the foundation for probing the complex processes of colloid transport and retention in stochastic nanoporous media. By adjusting simulation parameters, we successfully emulated various physicochemical environments. We carried out a systematic analysis of the impact of key factors, namely, *d*, Δ*P*, *E*_c–p_, and *E*_c–c_ on colloid transport dynamics, revealing the underlying mechanisms driving colloid retention. Notably, under conditions characterized by large *d*, low Δ*P*, and strong *E*_c–p_ attraction, we observed an atypical colloid jamming phenomenon. Our findings hold significant implications for understanding colloid transport in fine-grained geological systems. By shedding light on the intricate interplay of these fundamental factors, our research offers vital perspectives for improving the prediction and management of colloid transport and retention processes within such geological contexts. Furthermore, our work demonstrated that particle dynamics models can be used as a general framework for modeling and predicting colloidal behavior, and its generality makes it adaptable to diverse systems and materials. Moving forward, it is crucial to elucidate the influence of other pivotal factors, including colloid size, surface roughness, colloid hydrophobicity, and the nature of the porous structure, to refine predictions of colloid transport dynamics in nanoporous media.

## Supporting Information Available

Visualization of the colloid transport process and trajectories through a BNP medium (MOV)
Visualization of colloid retention by physical straining within nanoporous media (MOV)
Visualization of colloid retention by trapping in low-flow zone within nanoporous media (MOV)
Pressure effect on colloid transport in BNP media with scaled residence time by the pressure difference Δ*P* and system size effect on colloid transport in BNP media (PDF)

## Supplementary Material

la4c00037_si_001.mov

la4c00037_si_002.mov

la4c00037_si_003.mov

## Notes

The data that support the findings of this study is available from the corresponding author upon reasonable request.

## References

[ref1] Molnar I. L.; Pensini E.; Asad M. A.; Mitchell C. A.; Nitsche L. C.; Pyrak-Nolte L. J.; Miño G. L.; Krol M. M. Colloid Transport in Porous Media: A Review of Classical Mechanisms and Emerging Topics. Transp. Porous Media 2019, 130 (1), 129–156. 10.1007/s11242-019-01270-6.

[ref2] Petosa A. R.; Jaisi D. P.; Quevedo I. R.; Elimelech M.; Tufenkji N. Aggregation and Deposition of Engineered Nanomaterials in Aquatic Environments: Role of Physicochemical Interactions. Environ. Sci. Technol. 2010, 44 (17), 6532–6549. 10.1021/es100598h. 20687602

[ref3] Kumari A.; Yadav S. K.; Yadav S. C. Biodegradable Polymeric Nanoparticles Based Drug Delivery Systems. Colloids Surf., B 2010, 75 (1), 1–18. 10.1016/j.colsurfb.2009.09.001. 19782542

[ref4] O’Carroll D.; Sleep B.; Krol M.; Boparai H.; Kocur C. Nanoscale Zero Valent Iron and Bimetallic Particles for Contaminated Site Remediation. Adv. Water Resour. 2013, 51, 104–122. 10.1016/j.advwatres.2012.02.005.

[ref5] Yao K.-M.; Habibian M. T.; O’Melia C. R. Water and Waste Water Filtration. Concepts and Applications. Environ. Sci. Technol. 1971, 5 (11), 1105–1112. 10.1021/es60058a005.

[ref6] Molnar I. L.; Johnson W. P.; Gerhard J. I.; Willson C. S.; O’Carroll D. M. Predicting Colloid Transport through Saturated Porous Media: A Critical Review. Water Resour. Res. 2015, 51 (9), 6804–6845. 10.1002/2015WR017318.

[ref7] Frimmel F. H.; Von Der Kammer F.; Flemming H. C.. In Colloidal Transport in Porous Media; Frimmel F. H., Von Der Kammer F., Flemming H.-C., Eds.; Springer Berlin Heidelberg: Berlin, Heidelberg, 2007; . 10.1007/978-3-540-71339-5.

[ref8] Babakhani P.; Bridge J.; Doong R.; Phenrat T. Continuum-Based Models and Concepts for the Transport of Nanoparticles in Saturated Porous Media: A State-of-the-Science Review. Adv. Colloid Interface Sci. 2017, 246, 75–104. 10.1016/j.cis.2017.06.002. 28641812

[ref9] Rajagopalan R.; Tien C. Trajectory Analysis of Deep-Bed Filtration with the Sphere-in-Cell Porous Media Model. AIChE J. 1976, 22 (3), 523–533. 10.1002/aic.690220316.

[ref10] Tufenkji N.; Elimelech M. Correlation Equation for Predicting Single-Collector Efficiency in Physicochemical Filtration in Saturated Porous Media. Environ. Sci. Technol. 2004, 38 (2), 529–536. 10.1021/es034049r. 14750730

[ref11] Li X.; Li Z.; Zhang D. Role of Low Flow and Backward Flow Zones on Colloid Transport in Pore Structures Derived from Real Porous Media. Environ. Sci. Technol. 2010, 44 (13), 4936–4942. 10.1021/es903647g. 20540578

[ref12] Li Z.; Zhang D.; Li X. Tracking Colloid Transport in Porous Media Using Discrete Flow Fields and Sensitivity of Simulated Colloid Deposition to Space Discretization. Environ. Sci. Technol. 2010, 44 (4), 1274–1280. 10.1021/es9027716. 20088544

[ref13] Li X.; Lin C.-L.; Miller J. D.; Johnson W. P. Role of Grain-to-Grain Contacts on Profiles of Retained Colloids in Porous Media in the Presence of an Energy Barrier to Deposition. Environ. Sci. Technol. 2006, 40 (12), 3769–3774. 10.1021/es052501w. 16830540

[ref14] Johnson W. P.; Li X.; Yal G. Colloid Retention in Porous Media: Mechanistic Confirmation of Wedging and Retention in Zones of Flow Stagnation. Environ. Sci. Technol. 2007, 41 (4), 1279–1287. 10.1021/es061301x. 17593731

[ref15] Li X.; Lin C.-L.; Miller J. D.; Johnson W. P. Pore-Scale Observation of Microsphere Deposition at Grain-to-Grain Contacts over Assemblage-Scale Porous Media Domains Using X-Ray Microtomography. Environ. Sci. Technol. 2006, 40 (12), 3762–3768. 10.1021/es0525004. 16830539

[ref16] Shellenberger K.; Logan B. E. Effect of Molecular Scale Roughness of Glass Beads on Colloidal and Bacterial Deposition. Environ. Sci. Technol. 2002, 36 (2), 184–189. 10.1021/es015515k. 11827052

[ref17] Bhattacharjee S.; Ko C.-H.; Elimelech M. DLVO Interaction between Rough Surfaces. Langmuir 1998, 14 (12), 3365–3375. 10.1021/la971360b.

[ref18] Landkamer L. L.; Harvey R. W.; Scheibe T. D.; Ryan J. N. Colloid Transport in Saturated Porous Media: Elimination of Attachment Efficiency in a New Colloid Transport Model. Water Resour. Res. 2013, 49 (5), 2952–2965. 10.1002/wrcr.20195.

[ref19] Cornelis G. Fate Descriptors for Engineered Nanoparticles: The Good, the Bad, and the Ugly. Environ. Sci.: Nano 2015, 2 (1), 19–26. 10.1039/C4EN00122B.

[ref20] Bradford S. A.; Torkzaban S. Determining Parameters and Mechanisms of Colloid Retention and Release in Porous Media. Langmuir 2015, 31 (44), 12096–12105. 10.1021/acs.langmuir.5b03080. 26484563

[ref21] Cullen E.; O’Carroll D. M.; Yanful E. K.; Sleep B. Simulation of the Subsurface Mobility of Carbon Nanoparticles at the Field Scale. Adv. Water Resour. 2010, 33 (4), 361–371. 10.1016/j.advwatres.2009.12.001.

[ref22] Babakhani P.; Fagerlund F.; Shamsai A.; Lowry G. V.; Phenrat T. Modified MODFLOW-Based Model for Simulating the Agglomeration and Transport of Polymer-Modified Fe0 Nanoparticles in Saturated Porous Media. Environ. Sci. Pollut. Res. 2018, 25 (8), 7180–7199. 10.1007/s11356-015-5193-0. 26300356

[ref23] van Genuchten M. Th.; Wierenga P. J. Mass Transfer Studies in Sorbing Porous Media I. Analytical Solutions. Soil Sci. Soc. Am. J. 1976, 40 (4), 473–480. 10.2136/sssaj1976.03615995004000040011x.

[ref24] Dale A. L.; Casman E. A.; Lowry G. V.; Lead J. R.; Viparelli E.; Baalousha M. Modeling Nanomaterial Environmental Fate in Aquatic Systems. Environ. Sci. Technol. 2015, 49 (5), 2587–2593. 10.1021/es505076w. 25611674

[ref25] Seetha N.; Majid Hassanizadeh S.; Mohan Kumar M. S.; Raoof A. Correlation Equations for Average Deposition Rate Coefficients of Nanoparticles in a Cylindrical Pore. Water Resour. Res. 2015, 51 (10), 8034–8059. 10.1002/2015WR017723.

[ref26] Molnar I. L.; Sanematsu P. C.; Gerhard J. I.; Willson C. S.; O’Carroll D. M. Quantified Pore-Scale Nanoparticle Transport in Porous Media and the Implications for Colloid Filtration Theory. Langmuir 2016, 32 (31), 7841–7853. 10.1021/acs.langmuir.6b01233. 27385389

[ref27] de Vries E. T.; Tang Q.; Faez S.; Raoof A. Fluid Flow and Colloid Transport Experiment in Single-Porosity Sample; Tracking of Colloid Transport Behavior in a Saturated Micromodel. Adv. Water Resour. 2022, 159, 104086 10.1016/j.advwatres.2021.104086.

[ref28] Pan J.; Xiao S.; Zhang Z.; Wei N.; He J.; Zhao J. Nanoconfined Water Dynamics in Multilayer Graphene Nanopores. J. Phys. Chem. C 2020, 124 (32), 17819–17828. 10.1021/acs.jpcc.0c04897.

[ref29] Foroutan M.; Fatemi S. M.; Esmaeilian F. A Review of the Structure and Dynamics of Nanoconfined Water and Ionic Liquids via Molecular Dynamics Simulation. Eur. Phys. J. E 2017, 40 (2), 19 10.1140/epje/i2017-11507-7. 28229319

[ref30] Barsotti E.; Tan S. P.; Saraji S.; Piri M.; Chen J.-H. A Review on Capillary Condensation in Nanoporous Media: Implications for Hydrocarbon Recovery from Tight Reservoirs. Fuel 2016, 184, 344–361. 10.1016/j.fuel.2016.06.123.

[ref31] Majumder M.; Chopra N.; Andrews R.; Hinds B. J. Enhanced Flow in Carbon Nanotubes. Nature 2005, 438 (7064), 44 10.1038/438044a. 16267546

[ref32] Curtis M. E. Structural Characterization of Gas Shales on the Micro- and Nano-Scales. In All Days; SPE, 2010; Vol. 3, pp 1933–1947. 10.2118/137693-MS.

[ref33] Lubbers N.; Agarwal A.; Chen Y.; Son S.; Mehana M.; Kang Q.; Karra S.; Junghans C.; Germann T. C.; Viswanathan H. S. Modeling and Scale-Bridging Using Machine Learning: Nanoconfinement Effects in Porous Media. Sci. Rep. 2020, 10 (1), 13312 10.1038/s41598-020-69661-0. 32770012 PMC7414857

[ref34] Neil C. W.; Mehana M.; Hjelm R. P.; Hawley M. E.; Watkins E. B.; Mao Y.; Viswanathan H.; Kang Q.; Xu H. Reduced Methane Recovery at High Pressure Due to Methane Trapping in Shale Nanopores. Commun. Earth Environ. 2020, 1 (1), 49 10.1038/s43247-020-00047-w.

[ref35] Middleton R. S.; Carey J. W.; Currier R. P.; Hyman J. D.; Kang Q.; Karra S.; Jiménez-Martínez J.; Porter M. L.; Viswanathan H. S. Shale Gas and Non-Aqueous Fracturing Fluids: Opportunities and Challenges for Supercritical CO2. Appl. Energy 2015, 147, 500–509. 10.1016/j.apenergy.2015.03.023.

[ref36] Falk K.; Coasne B.; Pellenq R.; Ulm F.-J.; Bocquet L. Subcontinuum Mass Transport of Condensed Hydrocarbons in Nanoporous Media. Nat. Commun. 2015, 6 (1), 6949 10.1038/ncomms7949. 25901931 PMC4421809

[ref37] Thomas J. A.; McGaughey A. J. H. Reassessing Fast Water Transport Through Carbon Nanotubes. Nano Lett. 2008, 8 (9), 2788–2793. 10.1021/nl8013617. 18665654

[ref38] Joseph S.; Aluru N. R. Why Are Carbon Nanotubes Fast Transporters of Water?. Nano Lett. 2008, 8 (2), 452–458. 10.1021/nl072385q. 18189436

[ref39] Thomas J. A.; McGaughey A. J. H. Water Flow in Carbon Nanotubes: Transition to Subcontinuum Transport. Phys. Rev. Lett. 2009, 102 (18), 184502 10.1103/PhysRevLett.102.184502. 19518876

[ref40] Nicholls W. D.; Borg M. K.; Lockerby D. A.; Reese J. M. Water Transport through (7,7) Carbon Nanotubes of Different Lengths Using Molecular Dynamics. Microfluid. Nanofluid. 2012, 12 (1–4), 257–264. 10.1007/s10404-011-0869-3.

[ref41] Walther J. H.; Ritos K.; Cruz-Chu E. R.; Megaridis C. M.; Koumoutsakos P. Barriers to Superfast Water Transport in Carbon Nanotube Membranes. Nano Lett. 2013, 13 (5), 1910–1914. 10.1021/nl304000k. 23521014

[ref42] Wang S.; Javadpour F.; Feng Q. Fast Mass Transport of Oil and Supercritical Carbon Dioxide through Organic Nanopores in Shale. Fuel 2016, 181, 741–758. 10.1016/j.fuel.2016.05.057.

[ref43] Sui H.; Zhang F.; Wang Z.; Wang D.; Wang Y. Molecular Simulations of Oil Adsorption and Transport Behavior in Inorganic Shale. J. Mol. Liq. 2020, 305, 112745 10.1016/j.molliq.2020.112745.

[ref44] Wang S.; Javadpour F.; Feng Q. Molecular Dynamics Simulations of Oil Transport through Inorganic Nanopores in Shale. Fuel 2016, 171, 74–86. 10.1016/j.fuel.2015.12.071.

[ref45] Liang A.; Liu C.; Branicio P. S. Hot-Press Sintering of Aluminum Nitride Nanoceramics. Phys. Rev. Mater. 2021, 5 (9), 096001 10.1103/PhysRevMaterials.5.096001.

[ref46] Liang A.; Goodelman D. C.; Hodge A. M.; Farkas D.; Branicio P. S. CoFeNiTi and CrFeNiTi High Entropy Alloy Thin Films Microstructure Formation. Acta Mater. 2023, 257, 119163 10.1016/j.actamat.2023.119163.

[ref47] Hoogerbrugge P. J.; Koelman J. M. V. A. Simulating Microscopic Hydrodynamic Phenomena with Dissipative Particle Dynamics. Europhys. Lett. 1992, 19 (3), 155–160. 10.1209/0295-5075/19/3/001.

[ref48] Li Z.; Drazer G. Hydrodynamic Interactions in Dissipative Particle Dynamics. Phys. Fluids 2008, 20 (10), 103601 10.1063/1.2980039.

[ref49] Zhao J.; Chen S.; Zhang K.; Liu Y. A Review of Many-Body Dissipative Particle Dynamics (MDPD): Theoretical Models and Its Applications. Phys. Fluids 2021, 33 (11), 112002 10.1063/5.0065538.

[ref50] Bolintineanu D. S.; Grest G. S.; Lechman J. B.; Pierce F.; Plimpton S. J.; Schunk P. R. Particle Dynamics Modeling Methods for Colloid Suspensions. Comput. Part. Mech. 2014, 1 (3), 321–356. 10.1007/s40571-014-0007-6.

[ref51] Pan W.; Tartakovsky A. M. Dissipative Particle Dynamics Model for Colloid Transport in Porous Media. Adv. Water Resour. 2013, 58, 41–48. 10.1016/j.advwatres.2013.04.004.

[ref52] Xia Y.; Goral J.; Huang H.; Miskovic I.; Meakin P.; Deo M. Many-Body Dissipative Particle Dynamics Modeling of Fluid Flow in Fine-Grained Nanoporous Shales. Phys. Fluids 2017, 29 (5), 056601 10.1063/1.4981136.

[ref53] Xia Y.; Rao Q.; Hamed A.; Kane J.; Semeykina V.; Zharov I.; Deo M.; Li Z. Flow Reduction in Pore Networks of Packed Silica Nanoparticles: Insights from Mesoscopic Fluid Models. Langmuir 2022, 38 (26), 8135–8152. 10.1021/acs.langmuir.2c01038. 35731695

[ref54] Liu C.; Branicio P. S. Pore Size Dependence of Permeability in Bicontinuous Nanoporous Media. Langmuir 2021, 37 (51), 14866–14877. 10.1021/acs.langmuir.1c02615. 34902977

[ref55] da Silva G. C. Q.; Giro R.; Horta B. A. C.; Neumann R. F.; Engel M.; Steiner M. B. Effects of Additives on Oil Displacement in Nanocapillaries: A Mesoscale Simulation Study. J. Mol. Liq. 2020, 312, 112953 10.1016/j.molliq.2020.112953.

[ref56] Liu M.; Meakin P.; Huang H. Dissipative Particle Dynamics Simulation of Pore-Scale Multiphase Fluid Flow. Water Resour. Res. 2007, 43 (4), 1–14. 10.1029/2006WR004856.

[ref57] Xia Y.; Blumers A.; Li Z.; Luo L.; Tang Y.-H.; Kane J.; Goral J.; Huang H.; Deo M.; Andrew M. A GPU-Accelerated Package for Simulation of Flow in Nanoporous Source Rocks with Many-Body Dissipative Particle Dynamics. Comput. Phys. Commun. 2020, 247, 106874 10.1016/j.cpc.2019.106874.

[ref58] Pan W.; Caswell B.; Karniadakis G. E. Rheology, Microstructure and Migration in Brownian Colloidal Suspensions. Langmuir 2010, 26 (1), 133–142. 10.1021/la902205x. 20038167

[ref59] Yuen D. A.; Dzwinel W.; Boryczko K. Mesoscopic Dynamics of Colloids Simulated with Dissipative Particle Dynamics and Fluid Particle Model. J. Mol. Model. 2002, 8 (1), 33–43. 10.1007/s00894-001-0068-3. 12111400

[ref60] Chatterjee A.; Heine D. R.; Rovelstad A. L.; Wu L.-M. Modeling the Rheology of Suspensions with High-Viscosity Solvents: A Predictive Multiscale Approach. Phys. Rev. E: Stat., Nonlinear, Soft Matter Phys. 2009, 80 (2), 021406 10.1103/PhysRevE.80.021406. 19792123

[ref61] Rao Q.; Xia Y.; Li J.; McConnell J.; Sutherland J.; Li Z. A Modified Many-Body Dissipative Particle Dynamics Model for Mesoscopic Fluid Simulation: Methodology, Calibration, and Application for Hydrocarbon and Water. Mol. Simul. 2021, 47 (4), 363–375. 10.1080/08927022.2021.1876233.

[ref62] Español P.; Warren P. B. Perspective: Dissipative Particle Dynamics. J. Chem. Phys. 2017, 146 (15), 150901 10.1063/1.4979514. 28433024

[ref63] Liu M. B.; Liu G. R.; Zhou L. W.; Chang J. Z. Dissipative Particle Dynamics (DPD): An Overview and Recent Developments. Arch. Comput. Methods Eng. 2015, 22 (4), 529–556. 10.1007/s11831-014-9124-x.

[ref64] Warren P. B. Vapor-Liquid Coexistence in Many-Body Dissipative Particle Dynamics. Phys. Rev. E: Stat., Nonlinear, Soft Matter Phys. 2003, 68 (6), 066702 10.1103/PhysRevE.68.066702. 14754350

[ref65] Chen C.; Lu K.; Li X.; Dong J.; Lu J.; Zhuang L. A Many-Body Dissipative Particle Dynamics Study of Fluid-Fluid Spontaneous Capillary Displacement. RSC Adv. 2014, 4 (13), 6545 10.1039/c3ra47275b.

[ref66] Koelman J. M. V. A.; Hoogerbrugge P. J. Dynamic Simulations of Hard-Sphere Suspensions Under Steady Shear. Europhys. Lett. 1993, 21 (3), 363–368. 10.1209/0295-5075/21/3/018.

[ref67] Boek E. S.; Coveney P. V.; Lekkerkerker H. N. W.; van der Schoot P. Simulating the Rheology of Dense Colloidal Suspensions Using Dissipative Particle Dynamics. Phys. Rev. E: Stat. Phys., Plasmas, Fluids, Relat. Interdiscip. Top. 1997, 55 (3), 3124–3133. 10.1103/PhysRevE.55.3124.

[ref68] Boek E. S.; Coveney P. V.; Lekkerkerker H. N. W. Computer Simulation of Rheological Phenomena in Dense Colloidal Suspensions with Dissipative Particle Dynamics. J. Phys.: Condens. Matter 1996, 8 (47), 9509–9512. 10.1088/0953-8984/8/47/053.

[ref69] Li H.; Ye T.; Lam K. Y. Computational Analysis of Dynamic Interaction of Two Red Blood Cells in a Capillary. Cell Biochem. Biophys. 2014, 69 (3), 673–680. 10.1007/s12013-014-9852-4. 24590262

[ref70] Pan W.; Fedosov D. A.; Caswell B.; Karniadakis G. E. Predicting Dynamics and Rheology of Blood Flow: A Comparative Study of Multiscale and Low-Dimensional Models of Red Blood Cells. Microvasc. Res. 2011, 82 (2), 163–170. 10.1016/j.mvr.2011.05.006. 21640731 PMC3149761

[ref71] Ye T.; Phan-Thien N.; Lim C. T. Particle-Based Simulations of Red Blood Cells—A Review. J. Biomech. 2016, 49 (11), 2255–2266. 10.1016/j.jbiomech.2015.11.050. 26706718

[ref72] Ye T.; Phan-Thien N.; Khoo B. C.; Lim C. T. Dissipative Particle Dynamics Simulations of Deformation and Aggregation of Healthy and Diseased Red Blood Cells in a Tube Flow. Phys. Fluids 2014, 26 (11), 111902 10.1063/1.4900952.

[ref73] Hareendranath S.; Sathian S. P. Dynamic Response of Red Blood Cells in Health and Disease. Soft Matter 2023, 19 (6), 1219–1230. 10.1039/D2SM01090A. 36688330

[ref74] Liu Y.; Liu W. K. Rheology of Red Blood Cell Aggregation by Computer Simulation. J. Comput. Phys. 2006, 220 (1), 139–154. 10.1016/j.jcp.2006.05.010.

[ref75] Ju M.; Ye S. S.; Namgung B.; Cho S.; Low H. T.; Leo H. L.; Kim S. A Review of Numerical Methods for Red Blood Cell Flow Simulation. Comput. Methods Biomech. Biomed. Eng. 2015, 18 (2), 130–140. 10.1080/10255842.2013.783574. 23582050

[ref76] Pan W.; Fedosov D. A.; Karniadakis G. E.; Caswell B. Hydrodynamic Interactions for Single Dissipative-Particle-Dynamics Particles and Their Clusters and Filaments. Phys. Rev. E: Stat., Nonlinear, Soft Matter Phys. 2008, 78 (4), 046706 10.1103/PhysRevE.78.046706. 18999560

[ref77] Pryamitsyn V.; Ganesan V. A Coarse-Grained Explicit Solvent Simulation of Rheology of Colloidal Suspensions. J. Chem. Phys. 2005, 122 (10), 104906 10.1063/1.1860557. 15836357

[ref78] Dzwinel W.; Yuen D. A. A Two-Level, Discrete-Particle Approach for Simulating Ordered Colloidal Structures. J. Colloid Interface Sci. 2000, 225 (1), 179–190. 10.1006/jcis.2000.6751. 10767158

[ref79] Verwey E. J. W. Theory of the Stability of Lyophobic Colloids. J. Phys. Colloid Chem. 1947, 51 (3), 631–636. 10.1021/j150453a001. 20238663

[ref80] Derjaguin B.; Landau L. Theory of the Stability of Strongly Charged Lyophobic Sols and of the Adhesion of Strongly Charged Particles in Solutions of Electrolytes. Prog. Surf. Sci. 1993, 43 (1–4), 30–59. 10.1016/0079-6816(93)90013-L.

[ref81] Hamaker H. C. The London—van Der Waals Attraction between Spherical Particles. Physica 1937, 4 (10), 1058–1072. 10.1016/S0031-8914(37)80203-7.

[ref82] Everaers R.; Ejtehadi M. R. Interaction Potentials for Soft and Hard Ellipsoids. Phys. Rev. E: Stat., Nonlinear, Soft Matter Phys. 2003, 67 (4), 041710 10.1103/PhysRevE.67.041710. 12786380

[ref83] Safran S. A. Statistical Thermodynamics of Surfaces, Interfaces, and Membranes; CRC Press, 2018; . 10.1201/9780429497131.

[ref84] Santo K. P.; Neimark A. V. Dissipative Particle Dynamics Simulations in Colloid and Interface Science: A Review. Adv. Colloid Interface Sci. 2021, 298 (October), 102545 10.1016/j.cis.2021.102545. 34757286

[ref85] Liu C.; Branicio P. S. Efficient Generation of Non-Cubic Stochastic Periodic Bicontinuous Nanoporous Structures. Comput. Mater. Sci. 2019, 169, 109101 10.1016/j.commatsci.2019.109101.

[ref86] Hodge A. M.; Hayes J. R.; Caro J. A.; Biener J.; Hamza A. V. Characterization and Mechanical Behavior of Nanoporous Gold. Adv. Eng. Mater. 2006, 8 (9), 853–857. 10.1002/adem.200600079.

[ref87] Weissmüller J.; Newman R. C.; Jin H.-J.; Hodge A. M.; Kysar J. W. Nanoporous Metals by Alloy Corrosion: Formation and Mechanical Properties. MRS Bull. 2009, 34 (8), 577–586. 10.1557/mrs2009.157.

[ref88] Yeh W. J.; Chava S. Fabrication of Metallic Nanoporous Films by Dealloying. J. Vac. Sci. Technol. B 2009, 27 (2), 923–927. 10.1116/1.3032903.

[ref89] Erlebacher J.; Aziz M. J.; Karma A.; Dimitrov N.; Sieradzki K. Evolution of Nanoporosity in Dealloying. Nature 2001, 410 (6827), 450–453. 10.1038/35068529. 11260708

[ref90] Enke D.; Janowski F.; Schwieger W. Porous Glasses in the 21st Century—-a Short Review. Microporous Mesoporous Mater. 2003, 60 (1–3), 19–30. 10.1016/S1387-1811(03)00329-9.

[ref91] Zhao J.; Yao J.; Zhang M.; Zhang L.; Yang Y.; Sun H.; An S.; Li A. Study of Gas Flow Characteristics in Tight Porous Media with a Microscale Lattice Boltzmann Model. Sci. Rep. 2016, 6 (1), 32393 10.1038/srep32393. 27587293 PMC5009359

[ref92] Raeini A. Q.; Blunt M. J.; Bijeljic B. Direct Simulations of Two-Phase Flow on Micro-CT Images of Porous Media and Upscaling of Pore-Scale Forces. Adv. Water Resour. 2014, 74, 116–126. 10.1016/j.advwatres.2014.08.012.

[ref93] Goral J.; Panja P.; Deo M.; Andrew M.; Linden S.; Schwarz J.-O.; Wiegmann A. Confinement Effect on Porosity and Permeability of Shales. Sci. Rep. 2020, 10 (1), 49 10.1038/s41598-019-56885-y. 31913330 PMC6949243

[ref94] Thompson A. P.; Aktulga H. M.; Berger R.; Bolintineanu D. S.; Brown W. M.; Crozier P. S.; in ’t Veld P. J.; Kohlmeyer A.; Moore S. G.; Nguyen T. D.; Shan R.; Stevens M. J.; Tranchida J.; Trott C.; Plimpton S. J. LAMMPS - a Flexible Simulation Tool for Particle-Based Materials Modeling at the Atomic, Meso, and Continuum Scales. Comput. Phys. Commun. 2022, 271, 108171 10.1016/j.cpc.2021.108171.

[ref95] Shire T.; Hanley K. J.; Stratford K. DEM Simulations of Polydisperse Media: Efficient Contact Detection Applied to Investigate the Quasi-Static Limit. Comput. Part. Mech. 2021, 8 (4), 653–663. 10.1007/s40571-020-00361-2.

[ref96] Li Z.; Hu G.-H.; Wang Z.-L.; Ma Y.-B.; Zhou Z.-W. Three Dimensional Flow Structures in a Moving Droplet on Substrate: A Dissipative Particle Dynamics Study. Phys. Fluids 2013, 25 (7), 072103 10.1063/1.4812366.

[ref97] in ’t Veld P. J.; Plimpton S. J.; Grest G. S. Accurate and Efficient Methods for Modeling Colloidal Mixtures in an Explicit Solvent Using Molecular Dynamics. Comput. Phys. Commun. 2008, 179 (5), 320–329. 10.1016/j.cpc.2008.03.005.

[ref98] Stukowski A. Visualization and Analysis of Atomistic Simulation Data with OVITO-the Open Visualization Tool. Modell. Simul. Mater. Sci. Eng. 2010, 18 (1), 015012 10.1088/0965-0393/18/1/015012.

[ref99] Fan D.; Chapman E.; Khan A.; Iacoviello F.; Mikutis G.; Pini R.; Striolo A. Anomalous Transport of Colloids in Heterogeneous Porous Media: A Multi-Scale Statistical Theory. J. Colloid Interface Sci. 2022, 617, 94–105. 10.1016/j.jcis.2022.02.127. 35272170

[ref100] Liu A. J.; Nagel S. R. Jamming Is Not Just Cool Any More. Nature 1998, 396 (6706), 21–22. 10.1038/23819.

[ref101] Dressaire E.; Sauret A. Clogging of Microfluidic Systems. Soft Matter 2017, 13 (1), 37–48. 10.1039/C6SM01879C. 27801463

[ref102] Cates M. E.; Wittmer J. P.; Bouchaud J.-P.; Claudin P. Jamming, Force Chains, and Fragile Matter. Phys. Rev. Lett. 1998, 81 (9), 1841–1844. 10.1103/PhysRevLett.81.1841.

[ref103] Majekodunmi O. T.; Hashmi S. M. Flow Dynamics through Discontinuous Clogs of Rigid Particles in Tapered Microchannels. Sci. Rep. 2022, 12 (1), 22587 10.1038/s41598-022-25831-w. 36585430 PMC9803713

[ref104] Stoop R. L.; Tierno P. Clogging and Jamming of Colloidal Monolayers Driven across Disordered Landscapes. Commun. Phys. 2018, 1 (1), 68 10.1038/s42005-018-0068-6.

[ref105] Haw M. D. Jamming, Two-Fluid Behavior, and “Self-Filtration” in Concentrated Particulate Suspensions. Phys. Rev. Lett. 2004, 92 (18), 185506 10.1103/PhysRevLett.92.185506. 15169501

[ref106] Genovese D.; Sprakel J. Crystallization and Intermittent Dynamics in Constricted Microfluidic Flows of Dense Suspensions. Soft Matter 2011, 7 (8), 3889 10.1039/c0sm01338b.

[ref107] Campbell A. I.; Haw M. D. Jamming and Unjamming of Concentrated Colloidal Dispersions in Channel Flows. Soft Matter 2010, 6 (19), 4688 10.1039/c0sm00110d.

[ref108] Linkhorst J.; Beckmann T.; Go D.; Kuehne A. J. C.; Wessling M. Microfluidic Colloid Filtration. Sci. Rep. 2016, 6 (1), 22376 10.1038/srep22376. 26927706 PMC4772133

[ref109] Johnson W. P.; Pazmino E.; Ma H. Direct Observations of Colloid Retention in Granular Media in the Presence of Energy Barriers, and Implications for Inferred Mechanisms from Indirect Observations. Water Res. 2010, 44 (4), 1158–1169. 10.1016/j.watres.2009.12.014. 20132959

[ref110] Wu T.; Yang Z.; Hu R.; Chen Y.-F. Three-Dimensional Visualization Reveals Pore-Scale Mechanisms of Colloid Transport and Retention in Two-Phase Flow. Environ. Sci. Technol. 2023, 57 (5), 1997–2005. 10.1021/acs.est.2c08757. 36602921

[ref111] Carstens J. F.; Bachmann J.; Neuweiler I. A New Approach to Determine the Relative Importance of DLVO and Non-DLVO Colloid Retention Mechanisms in Porous Media. Colloids Surf., A 2019, 560, 330–335. 10.1016/j.colsurfa.2018.10.013.

[ref112] Johnson W. P.; Ma H.; Pazmino E. Straining Credibility: A General Comment Regarding Common Arguments Used to Infer Straining As the Mechanism of Colloid Retention in Porous Media. Environ. Sci. Technol. 2011, 45 (9), 3831–3832. 10.1021/es200868e. 21446725

[ref113] Boutt D. F.; Grasselli G.; Fredrich J. T.; Cook B. K.; Williams J. R. Trapping Zones: The Effect of Fracture Roughness on the Directional Anisotropy of Fluid Flow and Colloid Transport in a Single Fracture. Geophys. Res. Lett. 2006, 33 (21), L21402 10.1029/2006GL027275.

[ref114] Tong M.; Johnson W. P. Excess Colloid Retention in Porous Media as a Function of Colloid Size, Fluid Velocity, and Grain Angularity. Environ. Sci. Technol. 2006, 40 (24), 7725–7731. 10.1021/es061201r. 17256519

[ref115] Torkzaban S.; Bradford S. A.; van Genuchten M. T.; Walker S. L. Colloid Transport in Unsaturated Porous Media: The Role of Water Content and Ionic Strength on Particle Straining. J. Contam. Hydrol. 2008, 96 (1–4), 113–127. 10.1016/j.jconhyd.2007.10.006. 18068262

[ref116] Bradford S. A.; Kim H. N.; Haznedaroglu B. Z.; Torkzaban S.; Walker S. L. Coupled Factors Influencing Concentration-Dependent Colloid Transport and Retention in Saturated Porous Media. Environ. Sci. Technol. 2009, 43 (18), 6996–7002. 10.1021/es900840d. 19806733

[ref117] Tufenkji N.; Elimelech M. Breakdown of Colloid Filtration Theory: Role of the Secondary Energy Minimum and Surface Charge Heterogeneities. Langmuir 2005, 21 (3), 841–852. 10.1021/la048102g. 15667159

[ref118] Kusova A. M.; Sitnitsky A. E.; Zuev Y. F. The Role of PH and Ionic Strength in the Attraction-Repulsion Balance of Fibrinogen Interactions. Langmuir 2021, 37 (34), 10394–10401. 10.1021/acs.langmuir.1c01803. 34403253

[ref119] Bizmark N.; Ioannidis M. A. Effects of Ionic Strength on the Colloidal Stability and Interfacial Assembly of Hydrophobic Ethyl Cellulose Nanoparticles. Langmuir 2015, 31 (34), 9282–9289. 10.1021/acs.langmuir.5b01857. 26241005

[ref120] Nishad S.; Al-Raoush R. I. Colloid Retention and Mobilization Mechanisms under Different Physicochemical Conditions in Porous Media: A Micromodel Study. Powder Technol. 2021, 377, 163–173. 10.1016/j.powtec.2020.08.086.

[ref121] Shang J.; Liu C.; Wang Z. Transport and Retention of Engineered Nanoporous Particles in Porous Media: Effects of Concentration and Flow Dynamics. Colloids Surf., A 2013, 417, 89–98. 10.1016/j.colsurfa.2012.10.030.

[ref122] Bizmark N.; Schneider J.; Priestley R. D.; Datta S. S. Multiscale Dynamics of Colloidal Deposition and Erosion in Porous Media. Sci. Adv. 2020, 6 (46), 1–11. 10.1126/sciadv.abc2530. PMC7673751 33188022

[ref123] Zhang W.; Morales V. L.; Cakmak M. E.; Salvucci A. E.; Geohring L. D.; Hay A. G.; Parlange J.-Y.; Steenhuis T. S. Colloid Transport and Retention in Unsaturated Porous Media: Effect of Colloid Input Concentration. Environ. Sci. Technol. 2010, 44 (13), 4965–4972. 10.1021/es100272f. 20521810

[ref124] Laganapan A. M.; Mouas M.; Videcoq A.; Cerbelaud M.; Bienia M.; Bowen P.; Ferrando R. How Colloid-Colloid Interactions and Hydrodynamic Effects Influence the Percolation Threshold: A Simulation Study in Alumina Suspensions. J. Colloid Interface Sci. 2015, 458, 241–246. 10.1016/j.jcis.2015.07.058. 26232284

[ref125] Sang W.; Morales V. L.; Zhang W.; Stoof C. R.; Gao B.; Schatz A. L.; Zhang Y.; Steenhuis T. S. Quantification of Colloid Retention and Release by Straining and Energy Minima in Variably Saturated Porous Media. Environ. Sci. Technol. 2013, 47 (15), 130724151622003 10.1021/es400288c. 23805840

[ref126] Sirivithayapakorn S.; Keller A. Transport of Colloids in Saturated Porous Media: A Pore-Scale Observation of the Size Exclusion Effect and Colloid Acceleration. Water Resour. Res. 2003, 39 (4), 1109 10.1029/2002WR001583.

[ref127] Torkzaban S.; Bradford S. A. Critical Role of Surface Roughness on Colloid Retention and Release in Porous Media. Water Res. 2016, 88, 274–284. 10.1016/j.watres.2015.10.022. 26512805

[ref128] Auset M.; Keller A. A. Pore-Scale Visualization of Colloid Straining and Filtration in Saturated Porous Media Using Micromodels. Water Resour. Res. 2006, 42 (12), 1–9. 10.1029/2005WR004639.

[ref129] Bradford S. A.; Kim H.; Shen C.; Sasidharan S.; Shang J. Contributions of Nanoscale Roughness to Anomalous Colloid Retention and Stability Behavior. Langmuir 2017, 33 (38), 10094–10105. 10.1021/acs.langmuir.7b02445. 28846425

[ref130] Li T.; Shen C.; Wu S.; Jin C.; Bradford S. A. Synergies of Surface Roughness and Hydration on Colloid Detachment in Saturated Porous Media: Column and Atomic Force Microscopy Studies. Water Res. 2020, 183, 116068 10.1016/j.watres.2020.116068. 32619803

[ref131] Zhang Q.; Hassanizadeh S. M.; Liu B.; Schijven J. F.; Karadimitriou N. K. Effect of Hydrophobicity on Colloid Transport during Two-phase Flow in a Micromodel. Water Resour. Res. 2014, 50 (10), 7677–7691. 10.1002/2013WR015198.

[ref132] Du Q.; Zhou P.; Pan Y.; Qu X.; Liu L.; Yu H.; Hou J. Influence of Hydrophobicity and Roughness on the Wetting and Flow Resistance of Water Droplets on Solid Surface: A Many-Body Dissipative Particle Dynamics Study. Chem. Eng. Sci. 2022, 249, 117327 10.1016/j.ces.2021.117327.

[ref133] Li Y.; Zhang H.; Bao M.; Wang Z. Dissipative Particle Dynamics Simulation on the Association between Polymer and Surfactant: Effects of Surfactant and Polymer Feature. Comput. Mater. Sci. 2012, 63, 154–162. 10.1016/j.commatsci.2012.06.007.

[ref134] Knappenberger T.; Flury M.; Mattson E. D.; Harsh J. B. Does Water Content or Flow Rate Control Colloid Transport in Unsaturated Porous Media?. Environ. Sci. Technol. 2014, 48 (7), 3791–3799. 10.1021/es404705d. 24588072

