# Supplementary material for: Colloid Transport in Bicontinuous Nanoporous Media
Source: Langmuir. 2024 May 17;40(21):10868–83. doi: 10.1021/acs.langmuir.4c00037 (PMC11140755; doi:10.1021/acs.langmuir.4c00037)
Supplement: Supplementary file 4 — la4c00037_si_004.pdf [file la4c00037_si_004.pdf]

Supporting Information for

Colloid Transport in Bicontinuous Nanoporous Media

Aoyan Liang, Chang Liu, and Paulo S. Branicio[*]

Mork Family Department of Chemical Engineering and Materials Science, University of Southern California, Los Angeles, CA 90089-0242, United States

Number of pages: 4

Number of figures: 2

Number of video files: 3.

[*] branicio@usc.edu

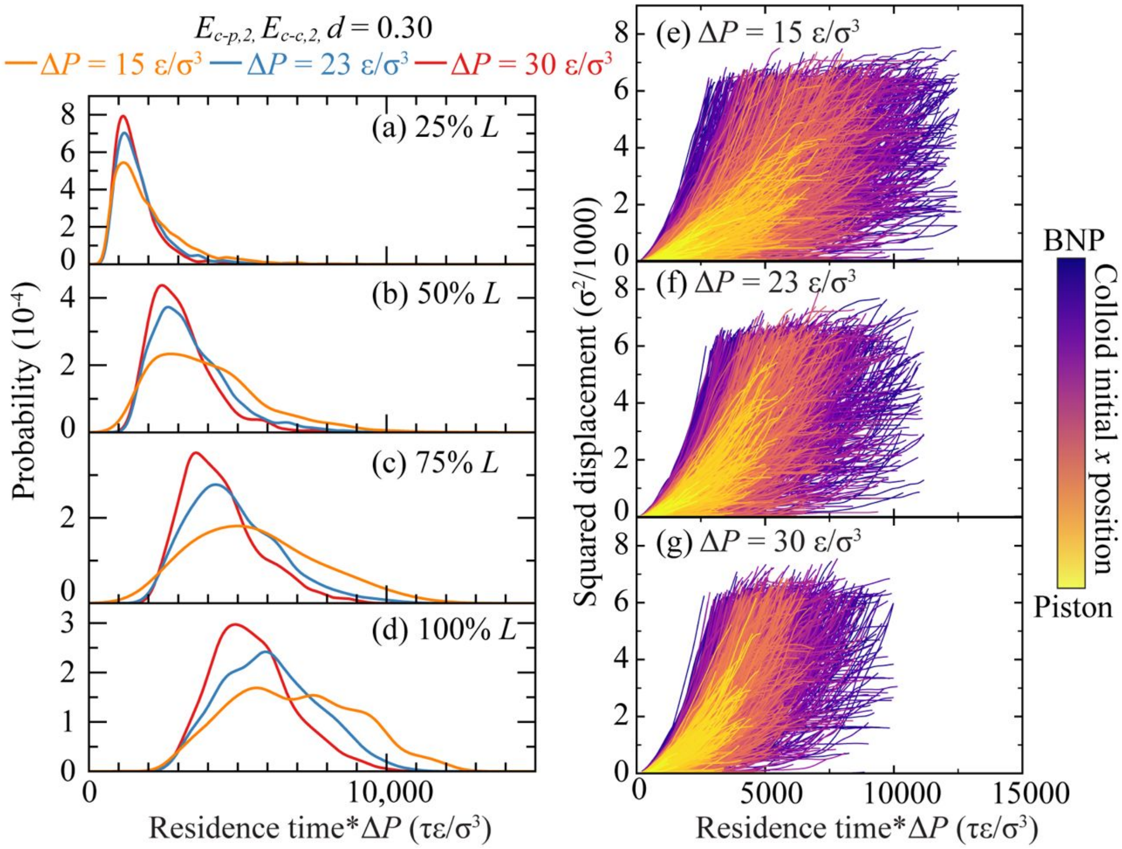

Figure S1. Pressure effect on colloid transport in BNP media with scaled residence time by the pressure difference ΔP. (a-d) Breakthrough curves when colloids breakthrough $25\% - 100\%\ L$ of the BNP media. Different colors represent different pressure difference, $\Delta P = 15$ to $30\ \varepsilon/\sigma^3$ while $E_{c-p} = E_{c-p,2}$, $E_{c-c} = E_{c-c,2}$, and $d = 0.30$. (e-g) Squared displacement of each colloid at $\Delta P = 15$, 23, and $30\ \varepsilon/\sigma^3$ as a function of the product of residence time and ΔP. Curves are colored by colloids initial positions along the x direction.

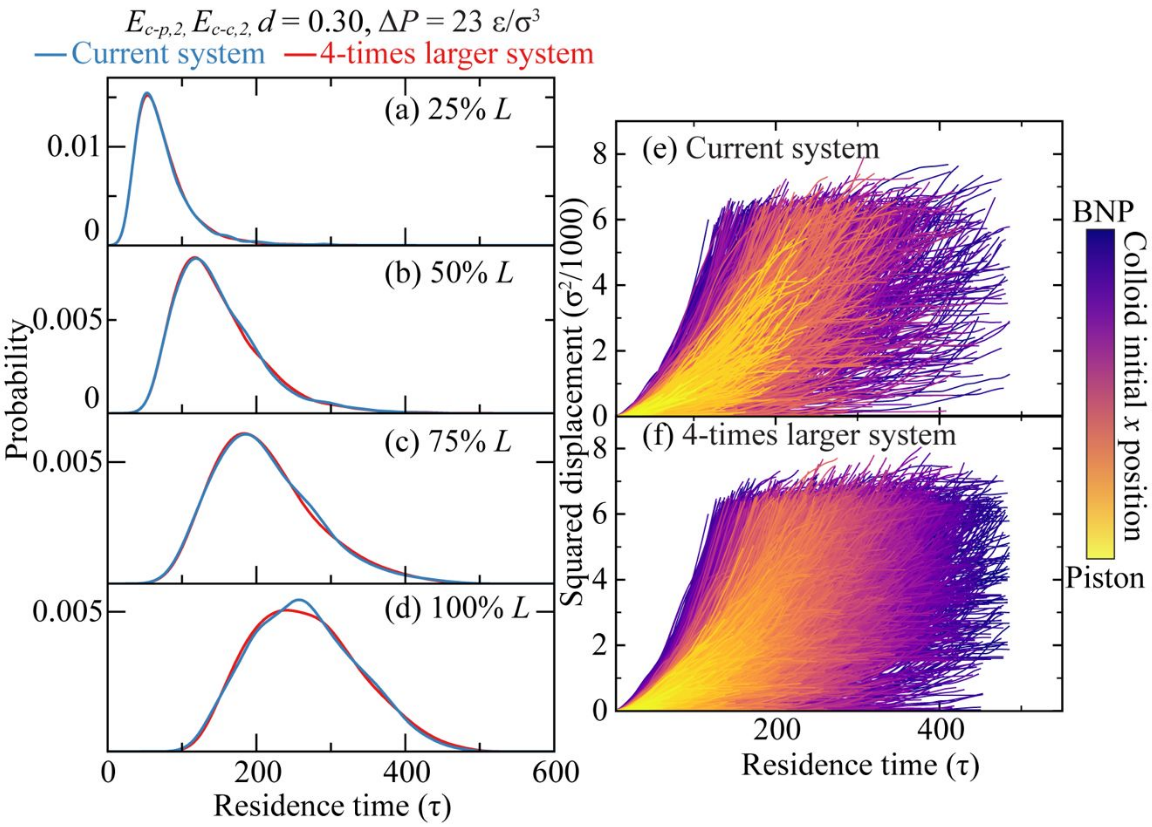

Figure S2. System size effect on colloid transport in BNP media. (a-d) Breakthrough curves when colloids breakthrough $25\% - 100\%\ L$ of the BNP media. Different colors represent different system sizes, while $\Delta P = 23\ \varepsilon/\sigma^3$ $E_{c-p} = E_{c-p,2}$, $E_{c-c} = E_{c-c,2}$, and $d = 0.30$. (e-g) Squared displacement of each colloid in our current simulation system and larger system as a function of residence time. Curves are colored by colloids initial positions along the x direction.

Movie S1. Colloid transport process and trajectories through a bicontinuous nanoporous medium.

Movie S2. Colloid retention within nanoporous media. The red colloid highlights the colloid retention by physical straining.

Movie S3. Colloid retention within nanoporous media. The red colloid highlights the colloid retention by trapping in low flow zone.
